# Manipulation of Chemistry and Biology with Visible Light Using Tetra‐*ortho*‐Substituted Azobenzenes and Azonium Ions

**DOI:** 10.1002/anie.202423506

**Published:** 2025-04-10

**Authors:** Francesca Cardano, Rosa Márquez García, Wiktor Szymanski

**Affiliations:** ^1^ Department of Chemistry University of Torino Via P. Giuria 7 Torino 10125 Italy; ^2^ Department of Medicinal Chemistry Photopharmacology and Imaging Groningen Research Institute of Pharmacy University of Groningen Groningen 9713 AV The Netherlands; ^3^ Department of Radiology Medical Imaging Center University of Groningen, University Medical Center Groningen Groningen 9713 GZ The Netherlands

**Keywords:** Azonium ions, Molecular photoswitches, Photochromism, Tetra‐*ortho*‐azobenzenes, Visible light

## Abstract

Molecular photoswitches are used for precise and reversible control over the properties and function of chemical, biological and material systems, offering exceptional spatiotemporal control. Their current development focuses on enabling operation with non‐damaging and deep tissue penetrating visible/near‐IR light. In this context, tetra‐*ortho*‐substituted azobenzenes and azonium ions play a leading role, thanks to their unique photophysical properties and easily modifiable structure. However, it is only recently that synthetic approaches to those sterically demanding systems have been established and their structure‐photochemistry relations have been understood to provide general rules for their tuning to a given application. In this review, we provide a comprehensive overview of this family of molecular photoswitches, providing an analysis of their photophysical properties, followed by a discussion of the available synthetic methodologies. Finally, we showcase the versatility of tetra‐*ortho*‐substituted azobenzenes and azonium ions for enabling light‐control in biological and material sciences, providing multiple insights for future applications.

## Graphical abstract

**Tetra‐*ortho*‐Azobenzenes (TOABs) in broad daylight**: in recent years, TOAB photoswitches became indispensable tools for all‐visible‐light manipulation of chemistry, biology and materials. This review explores their unique photochemical properties, design and synthetic approaches, offering support in choosing a TOAB for a given application.

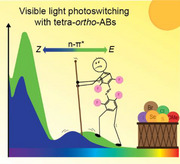

## 1 Introduction

### 1.1 Fundamental Principles of Molecular Photoswitch Operation

Light has been recognized as a precise, non‐invasive external stimulus for controlling chemical structures and processes.^[^ ^1^, ^2^, ^3^, ^4^, ^5^, ^6^ ^]^ On the molecular level, it enables control that is tunable—in terms of intensity (photon flux) and energy (photon wavelength), and that can be translated to micro and macroscopic effects. The electromagnetic radiation in the wavelength region between 300 and 2500 nm is of the highest operational interest in (applied) chemistry. The selection of unique wavelengths within this energy range is efficient especially for 1) precise chemical bond formation (in processes such as photo‐dimerization, [2 + 2]/[4 + 2]/[4 + 4] cycloadditions, photoredox catalytic reactions, photoclick or radical reactions); 2) chemical bonds breaking (photocleavage, photoinduced radical cleavage); and 3) photo‐isomerization/photo‐cyclization phenomena.^[^ ^7^, ^8^ ^]^ In biomedical sciences, light is increasingly used for therapy and diagnosis,^[^ ^6^, ^9^, ^10^ ^]^ taking a significant step forward from its traditional use in ancient medicine (heliotherapy, dermatologic treatment).^[^ ^11^ ^]^ The understanding of the nature of light and its interactions with human body enables its application in laser surgery, photodynamic therapy, and optical imaging.^[^ ^9^ ^]^ Similarly, light interaction with materials contributed to the development of the next generation of light‐responsive constructs,^[^ ^4^, ^12^ ^]^ including photochromic lenses, functional polymers and hydrogels, light‐sensitive nanoparticles (NPs), liquid crystals and energy/information storage materials.^[^ ^12^, ^13^, ^14^ ^]^

Implementing light responsivity in molecular systems for applications that require reversible control hinges on the use of molecular photoswitches.^[^ ^15^, ^16^, ^17^ ^]^ Photoswitches are molecules that are capable of reversible interconversion between isomeric forms [usually a stable (S) and a metastable (MS) form] upon their exposure to light at specific wavelengths. This interconversion can take place through different isomerization mechanisms, such as *E*/*Z* isomerization [azobenzenes (ABs),^[^ ^18^ ^]^ azoheteroarenes and diazocines,^[^ ^19^ ^]^ Azo‐BF_2_,^[^ ^20^ ^]^ iminothioindoxyls,^[^ ^21^ ^]^ hemi(thio)indigos and Indigoids^[^ ^22^ ^]^], electrocyclization [Dihydropyrenes (DHP),^[^ ^23^ ^]^ Stilbenes,^[^ ^15^ ^]^ Fulgides^[^ ^16^ ^]^] or by a combination of those processes [Donor–Acceptor Stenhouse Adducts (DASAs),^[^ ^24^ ^]^ and Spiropyrans^[^ ^15^, ^16^ ^]^].

The conversion of a photoswitch from the S to the MS state is mediated by the absorption of a light of a certain wavelength (λ_1_). The rate of this process (k_1_) depends on the light intensity (I), the molar extinction coefficient (ε) at λ_1_ and quantum yield (QY) for the forward switching from S to MS (Φ^S→MS^). The QY can be defined as the number of molecules that undergo isomerization per number of photons of the specific wavelength (λ_1_) that were absorbed by the reactant in a given time.^[^ ^10^ ^]^


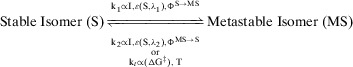




***Equation 1***. Photostationary equilibrium between the S and MS isomers.

The reverse transition can occur either photochemically, under irradiation with light of a different wavelength (λ_2_), or thermally. The rate (k_2_) of the photochemical back switching is governed by the light intensity (I), the molar extinction coefficient (ε) at λ_2_ and quantum yield for the back switching from MS to S (Φ^MS→S^). The rate (k_t_) for the thermal recovery depends on the activation energy barrier (ΔG^‡^) between the two isomers and the temperature. This process is characterized by a certain half‐life (*t*_1/2_ = ln(2)/k_t_) (Equation 1).^[^ ^10^ ^]^

Upon irradiation with λ_1_, over time, a photostationary state (PSS_(λ1)_) is established, where the rates of isomerization in both directions become equal. At PSS_(λ1)_, the metastable isomer is enriched, and this state is characterized by the photostationary state distribution (PSD) of the isomers.^[^ ^10^ ^]^ Upon irradiation with λ_2_, a different photostationary state (PSS_(λ2)_) is established where the solution is enriched in the stable isomer instead (Equation 2).^[^ ^16^ ^]^

$$\mathrm{PSD}={\left(\frac{\left[\text{MS}\right]}{\left[S\right]}\right)}_{\text{PSS}}=\frac{\phi_{S\to \mathrm{MS}}}{\phi_{\mathrm{MS}\to S}}\cdot \frac{\varepsilon_{S}}{\varepsilon_{\mathrm{MS}}}$$

***Equation 2***. Photostationary distribution (PSD) of isomers at PSS.

### 1.2 The Key Challenge of Using Low Energy Photons for Switching

**Figure 1:**
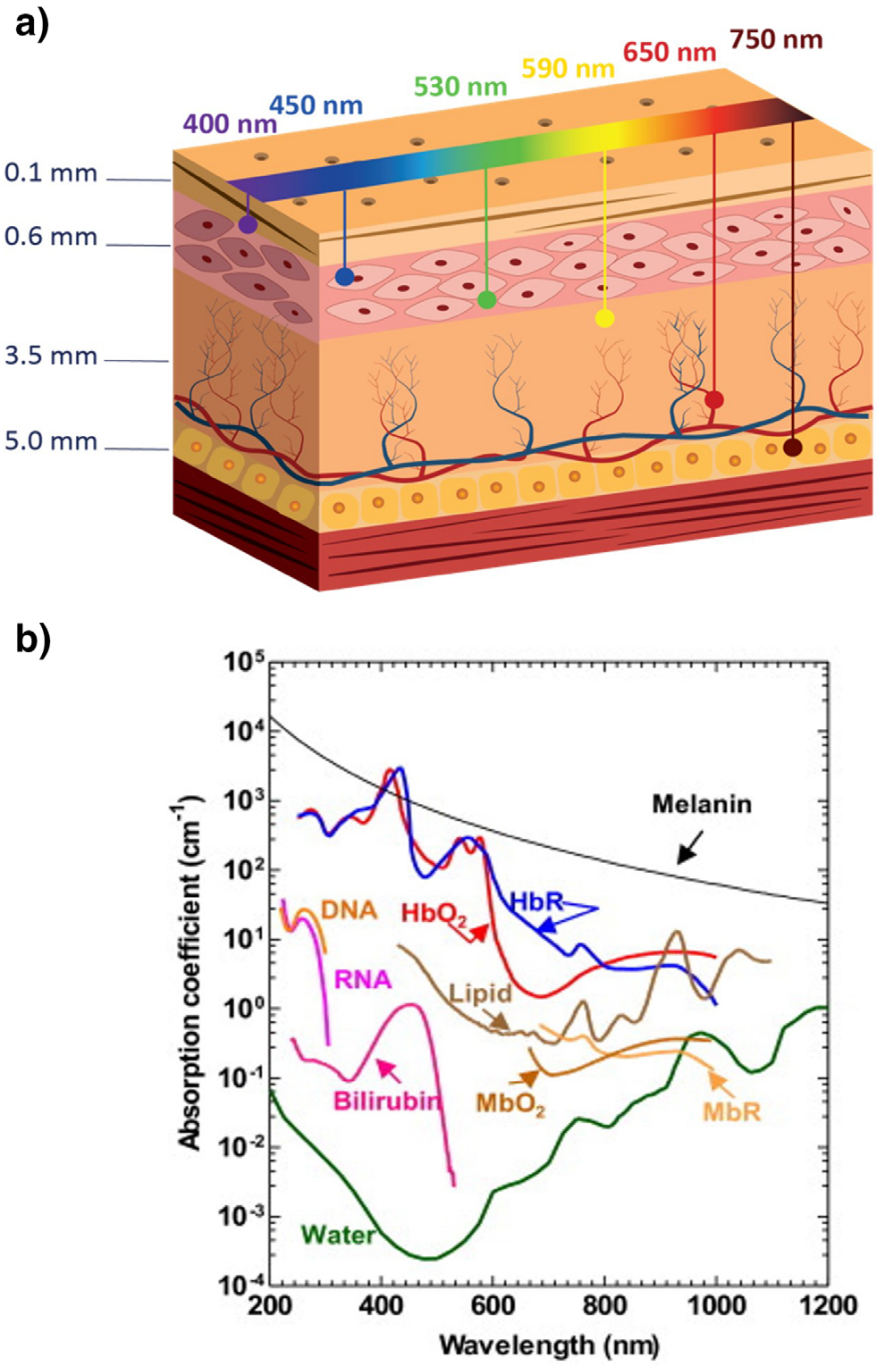
a) Tissue penetration depth of different light wavelengths. b) Absorption spectra of common endogenous contrast agents in biological tissue at physiological concentrations. Adapted with permission from ref. [26] Copyright © 2014 Elsevier.

The key challenge in designing and applying molecular photoswitches, especially in biological and material context where non‐destructive radiation and deep penetration is needed, is the requirement of UV light for switching in at least one of the directions.^[^ ^15^, ^16^ ^]^ For biomedical applications, the toxic effects of UV light pose a significant concern for human health,^[^ ^25^ ^]^ since UV light can induce mutagenic photoproducts or lesions in DNA.^[^ ^9^ ^]^ The use of visible and near‐infrared (NIR) light can address the intrinsic disadvantages of UV light, providing a safer (non‐toxic) alternative with enhanced penetration capabilities (Figure 1a).^[^ ^5^, ^12^ ^]^ While visible light overall presents a superior alternative to UV light, not all wavelengths within this region are equally effective in crossing tissues. In fact, the penetration depth varies significantly across wavelengths: UV and blue light (λ = 400–450 nm) typically penetrate only 0.1–0.4 mm, green and yellow light (500–590 nm) reach a depth of 0.4–1 mm, limited by melanin and hemoglobin absorption (Figure 1b). Red and NIR light (600–1.350 nm) achieve the deepest penetration, typically reaching depths of 2–5 mm into tissues. By using NIR wavelengths, both diagnostic accuracy and treatment outcomes can be improved in biomedical settings and deeper penetration can be obtained in material science.^[^ ^9^, ^10^, ^26^ ^]^

During the last decade, the advantages offered by visible/NIR light have driven the efforts in developing new families of photoswitches, aiming at the next generation of compounds with red‐shifted light‐responsivity, molecular diversity and robust applicability.^[^ ^5^, ^12^, ^27^, ^28^, ^29^ ^]^ In this context, ABs, while typically responsive to UV light,^[^ ^18^, ^28^ ^]^ served as the perfect starting point. Different strategies have been employed to render azobenzenes visible‐light‐responsive, such as the introduction of push‐pull systems,^[^ ^30^ ^]^ extension of the π‐conjugation, design of bridged ABs^[^ ^31^ ^]^ and tetra‐*ortho* substitution of AB core.^[^ ^32^, ^33^ ^]^

### 1.3 Tetra‐*ortho*‐azobenzenes as All‐visible Light Photoswitches

Tetra‐*ortho*‐ABs (TOABs), particularly those bearing substituents such as fluorine (‐F), chlorine (‐Cl) and methoxy (‐OCH₃), are extensively studied and widely utilized in applications that require switching solely by irradiation with visible light. Moreover, there is an increasing understanding on how the alterations in geometry and electronic configuration imparted by insertion of tetra‐*ortho*‐substitutions affect the intrinsic properties in comparison to the parent ABs.^[^ ^34^, ^35^, ^36^, ^37^ ^]^ Because of this, their overall photophysical traits can be easily tuned by combining *ortho* and *para* substituents on the AB core itself.

Tetra‐*ortho*‐methoxy‐substituted ABs [(MeO)_4_‐TOABs] hold a distinctive position within this class of compounds. Specifically, certain (MeO)_4_‐TOABs with amine‐type *para*‐substitutions have demonstrated the ability to be protonated and form the azonium ions under physiological pH (∼7) conditions.^[^ ^38^ ^]^ This phenomenon contrasts with other TOABs, which typically require much more acidic media for protonation.^[^ ^38^, ^39^ ^]^ The pH‐dependent behavior is noteworthy, as it enables precise control over molecular properties and functionalities, rendering these molecules particularly suitable for applications where pH sensitivity is advantageous.

**Figure 2:**
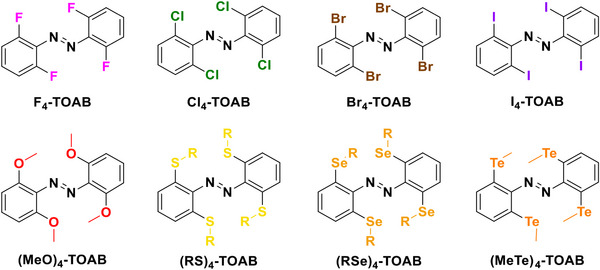
Overview on TOABs discussed in this review together with the color‐coding used.

This review focuses on TOABs (Figure 2) and azonium ions: two relatively young classes of molecules that have already found their applicational key role in several areas such as chemical biology, photopharmacology, polymer and material sciences making them tools‐of‐choice for visible light control. Herein, we outline their photochemical properties and delve into the available synthetic strategies, emphasizing their relevance in contemporary applications and discussing potential areas for improvement to guide future developments. Broader analyses focused on historical overview of ABs evolution^[^ ^32^ ^]^ or on applications of all visible light‐responsive ABs^[^ ^33^ ^]^ can also be found in the recent literature.

## 2 Photochemistry of TOABs

The parent azobenzene chromophore, composed of two benzene rings connected by a diazo bond, undergoes reversible *E/Z* isomerization, resulting in isomers with markedly distinct molecular geometries, which leads to unique photochemical and physical properties. The *trans (E)* isomer is typically thermodynamically stable (S), rigid and planar, while the *cis (Z)* isomer is metastable (MS), more flexible and bent, resulting in a significant difference in dipole moment (0 D vs. ∼3 D), shape (9 vs. 5.5 Å) and overall geometry.^[^ ^16^, ^18^, ^28^, ^40^ ^]^

The absorption spectrum of *E* isomer of the parent azobenzene (Figure 4a) exhibits two notable bands within the UV‐Vis region. The first is an intense band in UV range with a maximum wavelength (λ_max_) of approximately 310–320 nm and an extinction coefficient (ε) of around 22 000 L mol⁻¹ cm⁻¹, corresponding to a symmetry‐allowed π–π* transition. The second is a considerably weaker band in the visible region with ε of about 400 L mol⁻¹ cm⁻¹ and λ_max_ of approximately 440–450 nm, corresponding to the symmetry‐forbidden n–π* transition. The *Z* isomer shows two bands around 240 and 280 nm corresponding to the π–π* and a third one around 430 nm corresponding to the n–π* transition. The n–π* and π–π* transitions result in the excitation of azobenzene to its S_1_ and S_2_ states, respectively.^[^ ^16^, ^18^ ^]^

The parent azobenzene molecule not only shows an almost complete overlap of the n–π* transition bands of the *E* to the *Z* isomer, but also presents similar molar extinction coefficients (ε) of the *E* and *Z* isomers at this wavelength and higher back‐switching quantum yield (Φ*_Z_*_→_ *_E_ *= 0.46) than the forward one (Φ*_E_*_→_ *_Z_* = = 0.31).^[^ ^18^ ^]^ Consequently, when azobenzene is irradiated at wavelengths corresponding to the S_1_ (n–π*) transition, both isomers absorb light similarly, thereby preventing selective excitation and leading to inefficient photoconversion. As a result, the forward switching of conventional ABs is predominantly achieved via the excitation of the S_2_ (π–π*) transition, where there is a greater difference in the molar extinction coefficients between the *E* and *Z* isomers. Irradiation at this wavelength leads to efficient photoisomerization, typically yielding up to 80% of the *Z* isomer.^[^ ^18^ ^]^ While the *Z* isomer can be converted back to the *E* by irradiation at the S_1_ transition, this usually leads to poor *E* isomer content at PSS. In addition, the forward process is limited by the necessity of using UV light, which presents several drawbacks for applications.^[^ ^41^ ^]^

**Figure 3:**
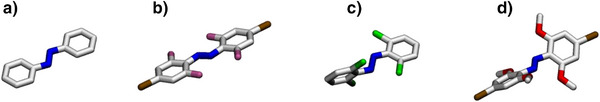
X‐ray crystal structure of a) parent AB;^[^ ^42^ ^]^ b) *para*‐bromo‐F_4_‐TOAB;^[^ ^43^ ^]^ c) Cl_4_‐TOAB;^[^ ^40^ ^]^ d) *para*‐bromo‐(MeO_4_)‐TOAB.^[^ ^44^ ^]^

A key strategy to address this limitation, used as the design principle of TOABs, involves the incorporation of four substituents in the *ortho* position of the diazo bond, which significantly impacts the molecule geometry and therefore the electronic distribution and photochemical properties. With the exception of fluorine, *ortho* substituents force the (*E*)‐AB molecule out of its planar geometry to minimize steric repulsive interactions by inducing the twisting of the phenyl rings out of the plane of the azo bond, as depicted in Figure 3.

**Figure 4:**
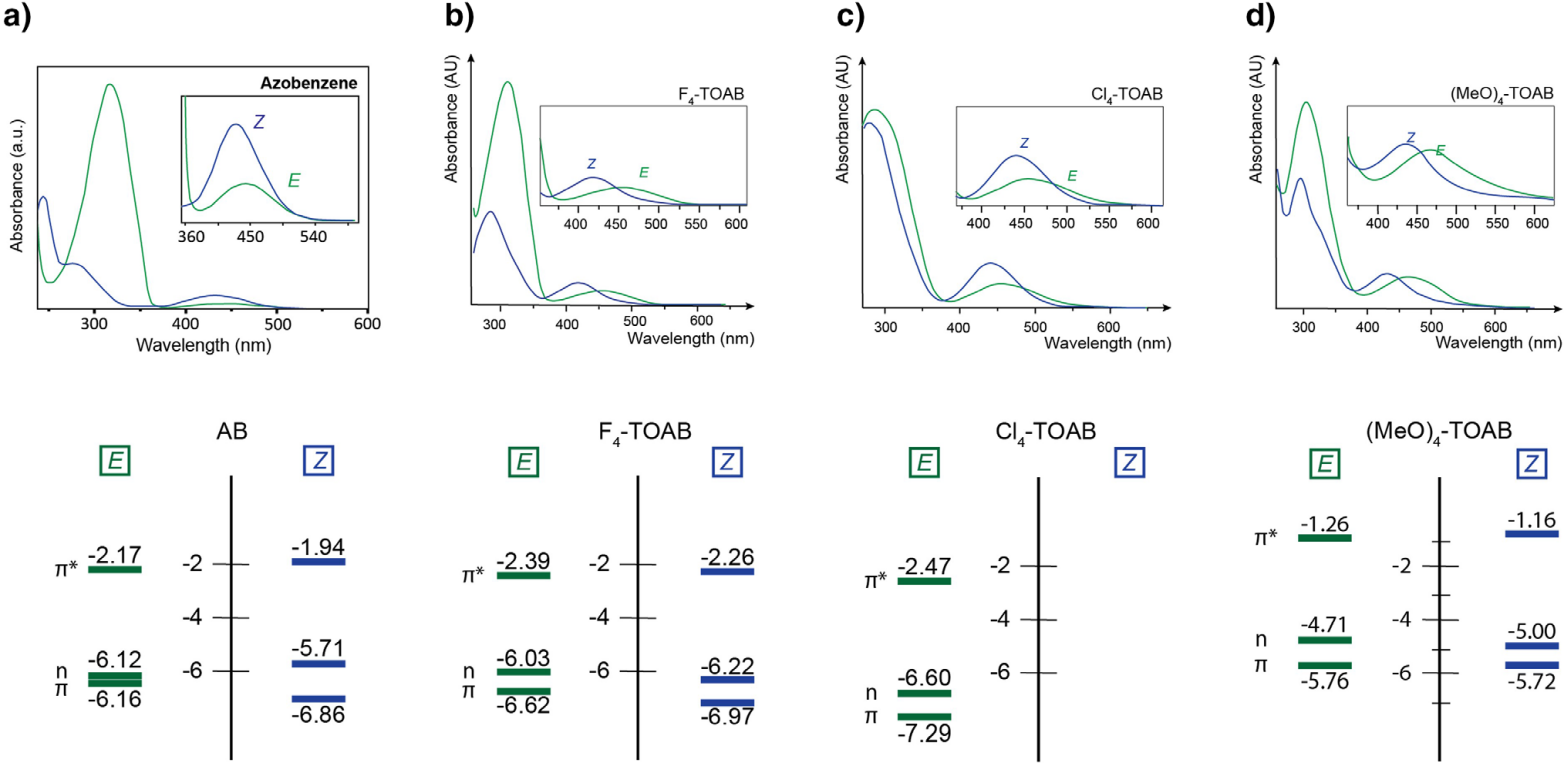
S_1_ transition band separation and energetic diagram of the π, n and π* orbitals of a) parent AB. Adapted with permission from ref. [35] Copyright © 2014 Wiley‐VCH Verlag GmbH & Co. KGaA. b) F_4_‐TOAB in DMSO at 25 °C; c) Cl_4_‐TOAB in DMSO at 37 °C; d) (MeO)_4_‐TOAB in DMSO at 37 °C.

This distortion, together with inductive and resonance effects introduced by the *ortho* substituents, affects the electron density distribution within the molecule and plays a crucial role in the stabilization or destabilization of the molecular orbitals (MOs) differently in each isomer (Figure 4). Therefore, *ortho* substituents allow for an increased separation between the isomers n–π* bands, thereby enabling to effectively switch solely with visible light (Table 1).^[^ ^40^, ^41^ ^]^

**Table 1:** Comparison of conventional TOABs properties.

						Φ^§^		
	Isomer	λ_max_ π–π* (nm)	λ_max_ n–π* (nm)	n–π* band separation	PSD %	S_2_	S_1_	*t*_1/2_
AB	*E*	317^a)^	444^a)^	14 nm	90^c)^	0.35^c)^	0.46^c)^	2 days^c)^
	*Z*	240^b)^ 280^b)^	430^a)^		80^c)^	0.15^c)^	0.31^c)^	
F_4_‐TOAB	*E*	314^d)^	460^d)^	43 nm	86^f)^	−	0.49^a)^	730 days^d)^
	*Z*	285^e)^	417^d)^		91^h)^	0.20^a)^	0.30^a)^	
Cl_4_‐TOAB	*E*	285^g)^	457^d)^	16 nm	85^i)^	−	0.48^d)^	38.1 days^d)^
	*Z*	280^g)^	441^d)^		90^j)^	−	0.51^d)^	
(MeO)_4_‐TOAB	*E*	305^k)^	475^k)^	45 nm	67^l)^	−	−	> 0.6 days^k)^
	*Z*	295^g)^	430^k)^		83^m)^	−	−	

^a)^ MeCN, 25 °C.^[^ ^35^ ^]^

^b)^ Estimated from a published spectrum.^[^ ^35^ ^]^

^c)^ ^[^ ^18^, ^47^ ^]^

^d)^ DMSO, 25 °C.^[^ ^37^ ^]^

^e)^ Estimated from a published.^[^ ^37^ ^]^

^f)^ DMSO, 25 °C, irradiation at 410 nm.^[^ ^41^ ^]^

^g)^ Estimated from a published spectrum.^[^ ^48^ ^]^

^h)^ DMSO, 25 °C, irradiation at > 450 nm.^[^ ^41^ ^]^

^i)^ DMSO, 25 °C, irradiation at 445 nm.^[^ ^37^, ^48^ ^]^

^j)^ DMSO, 25 °C, irradiation at 625 nm.^[^ ^37^, ^48^ ^]^

^k)^ DMSO, 37 °C.^[^ ^48^ ^]^

^l)^ DMSO, 37 °C, irradiation at 365 nm.^[^ ^48^ ^]^

^m)^ DMSO, 37 °C, irradiation at > 530 nm.^[^ ^48^ ^]^

^§^ *ϕ_E_* = *ϕ_Z→E_; ϕ_Z_* = *ϕ_E→Z_*

To introduce TOABs in applied molecular systems, different handles are introduced in their *para* position. These *para* substituents influence the electronic configuration of azobenzenes through direct conjugation with the aromatic system, exerting a pronounced effect on the π→π* transition. However, they also impact the n→π* transition by altering the electron density around the diazo nitrogen atoms. *Para* substituents play a pivotal role in properties such as absorption spectrum, photostationary state (PSS) and thermal stability of the *Z* isomer, which is directly related to its half‐life.^[^ ^37^, ^41^, ^45^, ^46^ ^]^ It is therefore of particular interest to understand the impact of *para* substitutions on the photochemical properties of TOABs.

In the following sections, we delve into the photochemical properties of various classes of TOABs (Figure 2), with a particular focus on how their substitution patterns influence these properties. By examining the relationship between structural modifications and photochemical behavior, we aim to provide a comprehensive understanding of the tunability of TOABs for specific applications.

### 2.1 *Ortho*‐halogen‐substituted TOABs

#### 2.1.1 Tetra‐*ortho*‐Fluoro‐Azobenzenes

The absorption spectrum of (*E*)‐F_4_‐TOAB displays two distinct bands: one at λ_max_ = 305 nm corresponding to the π–π* transition, and another at λ_max_ = 456 nm corresponding to the n–π* transition. The very small blue shift in the π–π* absorption compared to the parent unsubstituted compound suggests that the *E* isomer maintains near‐planarity,^[^ ^41^ ^]^ which is supported by the X‐ray crystal structure reported in Figure 3a.^[^ ^35^ ^]^ Regarding the S_1_ transition, the *E* isomer exhibits a +12 nm bathochromic shift relative to the parent azobenzene. Conversely, the same transition for the *Z* isomer is significantly hypsochromically shifted, showing a −16 nm difference compared to the parent azobenzene. The shifts in band positions relative to parent azobenzene (Figure 4b) can be explained considering that the inductive effects of the fluorine atoms stabilize 1) the π* orbital of both isomers;^[^ ^35^ ^]^ 2) the n‐MO of the *Z* isomer. For the *E* isomer, the stabilization of the π* orbital leads to a decrease in the energy of the n–π* transition, as shown in Figure 4b, which leads to a bathochromic shift of the band. On the other hand, the net effect of the combination of both effects in the *Z* isomer results in an increased energy gap for the n–π* transition, leading to an overall blue‐shift.^[^ ^35^, ^41^ ^]^ This results in a net 43 nm separation between the n–π* bands of the *E* and *Z* isomers. The quantum yields for the n–π* transitions of F_4_‐TOAB are comparable to those of unsubstituted azobenzene, with the QY for the back isomerization (Φ*_Z_*_→_ *_E _= *0.49) being higher than that of the forward process (Φ*_E_*_→_ *_Z _* = 0.30). Nevertheless, the pronounced band separation allows for high PSS ratios in the forward and back switching, reaching 86% of the *Z* isomer upon excitation at λ > 500 nm and 91% of the *E* isomer at λ = 410 nm.^[^ ^35^ ^]^

The *Z* form of F_4_‐TOAB is exceptionally stable, primarily due to the stabilizing effects of the σ‐EW (electron‐withdrawing) fluorine atoms located at the *ortho* positions. This stabilization is attributed to changes in the dipole moment of the *Z* isomer and the transition state during isomerization. The presence of σ‐EW fluorine atoms at the *ortho* positions contributes to a higher dipole moment in the *Z* isomer relative to the transition state, which leads to stronger stabilization of the *Z* isomer through dipole–dipole interactions in polar solvents. This increased stabilization raises the energy barrier for thermal isomerization, resulting in an unprecedented half‐life of 2 years in DMSO at 25 °C^[^ ^41^ ^]^ or 92 h in MeCN at 60 °C.^[^ ^35^ ^]^

F_4_‐TOABs substituted in one of the *para* positions with a σ‐EWG such as fluorine, bromine or an alkyne exhibit a larger bathochromic shift in the absorption of the n–π* transition of the *E* isomer compared to the *Z* isomer, relative to the *para*‐unsubstituted compound. This results in slightly broader band separations, ranging from 32 to 38 nm, compared to 30 nm for the *para*‐unsubstituted compound.^[^ ^46^ ^]^ When σ‐EWGs are linked in both *para* positions, the band separation remains consistent with that of the mono‐substituted compound.^[^ ^41^, ^46^ ^]^ EWGs in direct conjugation with the π‐system induce a redshift in the n–π* absorption of the *E* isomer. The λ_max_ values for substituents such as *bis*‐ester, *bis‐*cyano, *bis‐*amide^[^ ^35^ ^]^ or *bis*‐aldehyde^[^ ^49^ ^]^ are observed between 468 and 475 nm, compared to 456 nm for the *para*‐unsubstituted‐AB. It is noteworthy that this bathochromic shift is less pronounced when only one *para* position is substituted, resulting in a λ_max_ of 460 nm in the case of the ester^[^ ^35^ ^]^ or 466 nm for aldehyde.^[^ ^49^ ^]^ F_4_‐TOABs bearing π‐acceptor EWGs exhibit ∼ 50 nm separation of the n–π* bands of the *E* and *Z* isomers. This allows for almost complete photoconversion, resulting in high *Z* isomer content of 82%–92% under irradiation with λ > 500 nm and back isomerization 85%–97% *Z*→*E* when irradiated at ∼ 410 nm.^[^ ^41^, ^49^ ^]^ The substitution of both *para* positions of F_4_‐TOAB with two ester groups does not result in any change regarding the QY of the *Z* to *E* isomerization, Φ*_Z_*_→_ *_E _=* 0.49. However, the QY of the forward process is especially low for the *para‐*substituted compound (Φ*_E_*_→_ *_Z _* = 0.11), compared to Φ*_E_*_→_ *_Z _* = 0.30 for F_4_‐TOAB.

For F_4_‐TOABs with *para*‐EDGs (ED electron‐donating), excitation of the n–π* transition with light of λ > 500 nm effectively induces the *E→Z* isomerization, resulting in a PSS with 85% *Z* isomer content. However, due to the separation of the n–π* bands of the isomers reduced to 22 nm (compared to 42 nm for unsubstituted F_4_‐TOAB), the reverse isomerization leads to only 69% photoconversion to the *E* isomer when irradiated at 440 nm. The reduced band separation can be explained by the fact that EDGs in *para*‐positions raise the energy of the highest occupied MOs and counteract the effect of the σ‐electron‐withdrawing fluorine atoms, as indicated by quantum chemical calculations.^[^ ^41^ ^]^

The introduction of π‐acceptor groups at the *para* position significantly impacts the thermal stability of the *Z* isomer. For a better comparison of the influence of the *para* substitution on the half‐life, all data provided next were determined using MeCN as solvent at 60 °C. Under these conditions, F_4_‐TOAB has a half‐life of 92 h, while substituting π‐acceptor groups like esters^[^ ^35^ ^]^ or aldehydes at the *para* position reduces the half‐lives to 30 and 10.8 h, respectively.^[^ ^49^ ^]^ The thermal stability decreases further in doubly *para*‐substituted compounds, with bis‐amide exhibiting a half‐life of 22 h, bis‐ester 15 h, and bis‐aldehyde only ∼3 h.^[^ ^49^ ^]^ Quantum chemical calculations reproduce this observed trend by revealing how *para*‐substitution influences the energy levels of MOs. The incorporation of EDGs at the *para*‐position increases the thermal stability of the *Z* isomer by stabilizing the electronic structure. In contrast, the attachment of EWGs at the *para*‐position reduces the thermal stability of the *Z* isomer due to the destabilizing influence on the conjugated system.^[^ ^45^ ^]^ Therefore, the tendency outlined in the previous paragraph can be attributed to the π‐acceptor groups stabilizing the interactions of the doubly occupied p orbital on the inverting nitrogen atom in the linear transition state,^[^ ^35^, ^49^ ^]^ thus facilitating thermal isomerization.

Altogether, among all TOABs, the F_4_‐TOABs are characterized by a large band separation and exceptionally high thermal stability of the metastable isomer.

#### 2.1.2 Tetra‐*ortho*‐Chloro‐Aobenzenes

The absorption spectrum of (*E*)‐Cl_4_‐TOAB exhibits two distinct bands: the π–π* transition at λ_max_ = ∼280 nm and the n–π* transition at λ_max_ = 457 nm.^[^ ^37^ ^]^ The significant (−32 nm) hypsochromic shift in the π–π* absorption as compared to the parent azobenzene suggests that the steric bulk of the *ortho*‐chlorine atoms twists the two aryl rings of Cl_4_‐TOAB out of the plane, as confirmed by the X‐ray crystal structure (Figure 3b). At the same time, X‐ray crystallography also provides insight into the conformational flexibility of Cl_4_‐TOAB, which allows it to adopt different, red‐shifted geometries.^[^ ^40^ ^]^ The n–π* band of the (*E*)‐Cl_4_‐TOAB isomer is bathochromically shifted relative to the parent unsubstituted AB. This is explained by the inductive effect of chlorine atoms that stabilizes the π* orbitals of *E* isomer, reducing the energy required for the n–π* transition, similar to the effect observed with fluorine substituents.^[^ ^41^ ^]^ It can also be attributed to the repulsive interactions between the chlorine atoms and the lone pairs of the nitrogen atoms, which results in significant destabilization of the n MO of the *E* isomer. Consequently, the energy gap for the S₀–S₁ transition decreases, causing a bathochromic shift in the n–π* band.^[^ ^40^ ^]^ In the *Z*‐isomer, the repulsion is reduced, which causes a stabilization of the n‐MO of the *Z* isomer. Therefore, a hypsochromic shift of the n–π* band with λ_max_ = 441 nm can be observed, leading to 16 nm band separation between isomers.^[^ ^50^ ^]^ In contrast to parent AB, the QY for the n–π* transition of the forward switching (Φ*_E_*_→_ *_Z _* = 0.51) is comparable to that of the back isomerization (Φ*_Z_*_→_ *_E _= *0.48). This allows for high PSS ratios reaching 90% of the *Z* isomer upon excitation at λ = 625 nm and 85% of the *E* isomer at λ = 426 nm.

**Figure 5:**
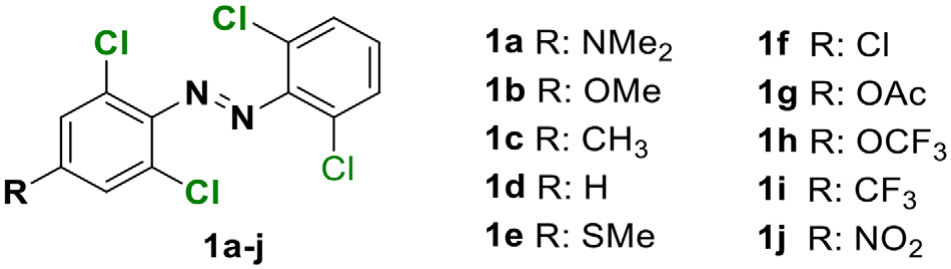
*Para*‐substituted Cl_4_‐TOABs analyzed in respect to the Hammet constant of *para* substituents.

With regard to the *para*‐position, both doubly substituted^[^ ^51^ ^]^ and mono substituted^[^ ^37^ ^]^ Cl_4_‐TOAB have been investigated (Figure 5). The effect of *para* substitution on the photochemical properties of Cl_4_‐TOAB correlates well with the substituent's Hammett constant. Regarding the QY, no general trends can be observed for different *para*‐substitution (Φ*_E_*_→_ *_Z _* = 38 ± 16%); only strong EDGs show somewhat higher QY than strong EWGs. For σ‐EDG and σ‐EWG, the n–π* transition band of the *E* isomer is observed in the range of λ = 444–461 nm, while the n–π* transition band of the *Z* isomer appears in the λ = 441–444 nm region. The *Z* isomer's n–π* band remains largely unchanged compared to the *para*‐unsubstituted compound, resulting in band separations of approximately 11–20 nm. This separation is similar to that observed in the *para*‐unsubstituted Cl_4_‐TOAB. As a result, photoconversions are also in the range typical for the *para*‐unsubstituted compound: red light irradiation yields 83%–90% *Z* isomer, while blue light irradiation produces 82%–90% *E* isomer.^[^ ^37^ ^]^

Compound **1a**, bearing a strong π‐EDG, does not present a well‐resolved n–π* transition band due to overlap with a strong π–π* band. Nonetheless, this substitution results in the most pronounced bathochromic shift in absorption. The overlap between the π–π* and n–π* bands is responsible for the inverted photoconversion behavior, where blue light induces switching to the *Z* isomer (66%), while green light induces switching to the *E* isomer (78%). On the other hand, in the case of π‐EW *para*‐substituted compound **1j**, even though it presents a promising band separation of 19 nm, the photoconversion efficiency for the forward switching is relatively low, since red light induces a PSS with only 45% of the *Z* isomer.^[^ ^37^ ^]^

This reduced efficiency in the forward switching is likely due to the relatively short half‐life of the *Z* isomer, where thermal back‐isomerization competes with the photochemical transformation, limiting the accumulation of the *Z* isomer. In fact, Cl_4_‐TOABs bearing groups such as NMe₂ (compound **1a**) and NO₂ (compound **1j**) in *para* position demonstrate significantly reduced *Z* isomer stability, with half‐lives on the order of hours. Even shorter half‐lives have been reported for the *para*‐disubstituted push‐pull Cl_4_‐TOAB, ranging in the order of milliseconds.^[^ ^51^ ^]^ However, this is an exception, since the half‐lives of the *Z* isomers for Cl_4_‐TOAB is 38 days, and for *para* mono‐substituted Cl_4_‐TOAB with mild σ‐EDG and σ‐EWG (compounds **1b**‐**1i**), it ranges from 4 to 32 days, which for most applications translates to bistable systems.^[^ ^37^ ^]^

Overall, despite presenting the smallest band separation among all TOABs, Cl_4_‐TOABs can be switched with red and blue light and exhibit the best QYs that enable reaching excellent PSDs.

#### 2.1.3 Tetra‐*ortho*‐Bromo‐Azobenzenes and Tetra‐*ortho*‐Iodo‐Azobenzenes

**Figure 6:**
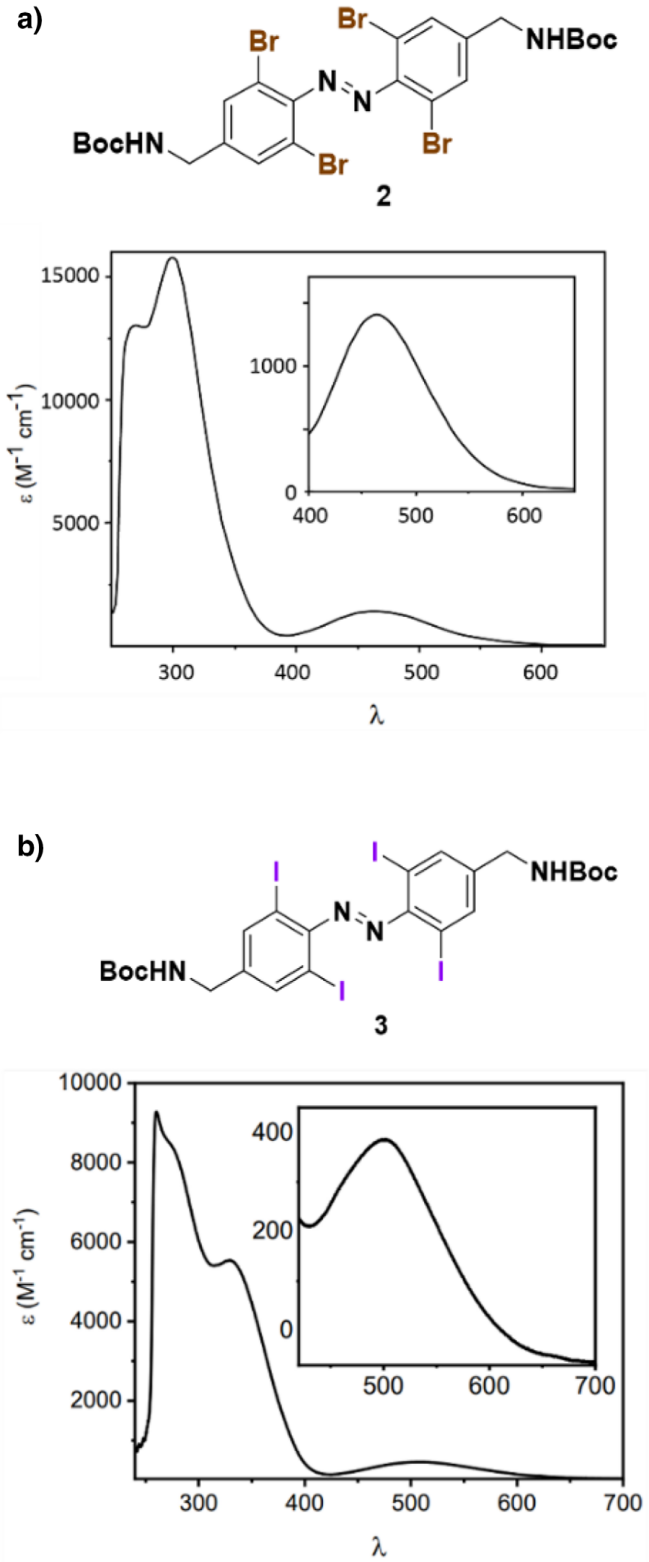
Absorption spectra of (a) compound **2**; b) compound **3**. Adapted with permission from ref. [50] Copyright © 2022 The Royal Society of Chemistry.

Heavier halogen atoms, such as bromine and iodine, induce significant bathochromic shifts in the n–π* absorption band of TOABs due to the destabilization of the n‐MO caused by the steric hindrance between the *ortho*‐substituent and the nitrogen lone pair. For Br_4_‐TOAB and I_4_‐TOAB bearing the same *para*‐substituent (i.e.*, para*‐CH_2_NHBoc, compounds **2** and **3**), the red‐shift is particularly pronounced in the case of the iodo derivative (Figure 6). This difference can be attributed to the larger atomic radius of iodine compared to bromine. (*E*)‐Br_4_‐TOAB shows a λ_max_ of 467 nm and tails up to 550 nm. It can be switched in the forward direction with 625 nm light, achieving a PSS containing up to 66% of the *Z* isomer. The bulkier (*E*)‐I_4_‐TOAB exhibits a λ_max_ at 514 nm with absorption band extending beyond 600 nm, and its photoisomerization can be achieved even using 660 nm light. However, PSS values have not been reported for **3**, and a representative PSS value has only been reported for the *para*‐CH2NPhth‐substituted I_4_‐TOAB, showing a maximum of 43% *Z* isomer under 660 nm irradiation. Additionally, for I_4_‐TOABs substituted with EWGs in *para* position, a slight bathochromic in the absorption can be observed, resulting in a λ_max_ of approximately 520 nm.^[^ ^50^ ^]^

With respect to the thermal back isomerization, there is a significant difference in the thermal stability of the *Z* isomer for Br_4_‐TOAB and I_4_‐TOAB. Compound **2** shows a half‐life of 12 h, whereas for compound **3,** it is only 5 min under the same conditions. This phenomenon can be attributed to the σ‐EW ability of the Br compared to I, since it contributes to the stabilization of the *Z* isomer.^[^ ^41^ ^]^ Moreover, π‐EWGs in *para* position of I_4_‐TOABs shorten the half‐lives down to the range of seconds.

Overall, TOABs substituted with heavy halogens exhibit significantly red‐shifted absorption spectra. In particular, I₄‐TOAB can be isomerized exclusively with far‐red light, as its half‐life can be precisely tuned to the range of seconds through modifications at the *para* position.

#### 2.1.4 Mixed Halogens

**Figure 7:**
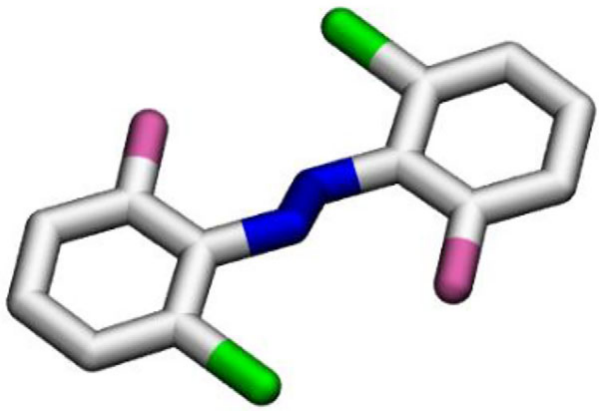
X‐ray crystal structure of nearly planar F_2_Cl_2_‐TOAB.^[^ ^40^ ^]^

Besides the homo‐substituted TOABs (with all four *ortho* substituents being the same), hetero‐substituted TOABs have also been investigated.^[^ ^37^, ^40^, ^52^, ^53^, ^54^ ^]^ Especially the combination of fluorine and chlorine *ortho*‐substituents in TOABs has been extensively studied.^[^ ^37^, ^40^ ^]^ (*E*)‐F_2_Cl_2_‐TOAB displays a more red‐shifted n–π* absorption band compared to the parent compounds (*E*)‐Cl_4_‐TOAB and (*E*)‐F_4_‐TOAB. This shift can be attributed to the near‐planar geometry (Figure 7) due to the reduced steric bulk of the *ortho* substituents, which allows for improved PSS values compared to Cl_4_‐TOAB when irradiated at 530 nm. Although F_2_‐Cl_2_‐TOAB requires prolonged irradiation times when switching with > 600 nm, it enables switching with up to 660 nm light, what cannot be achieved for F_4_‐TOAB.^[^ ^40^ ^]^

The thermal stability of the *Z* isomer remains comparable to that of the parent compound, F_4_‐TOAB. However, half‐life shortens upon the introduction of EWGs at the *para* position, despite remaining within the range of several hours at 25 °C.^[^ ^40^ ^]^

#### 2.1.5 Comparative Analysis of Tetra‐*ortho*‐Halogen ABs

**Figure 8:**
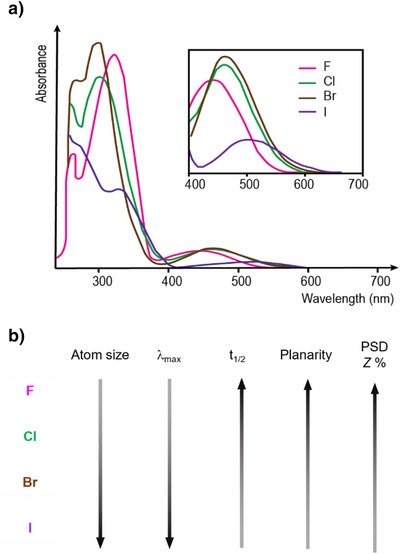
a) Absorption spectra of halogen‐substituted TOABs. Adapted with permission from ref. [50] Copyright © 2022 The Royal Society of Chemistry. b) General trends exhibited by key (photo)chemical properties of tetra‐*ortho*‐ABs with halogen substitution where PSD Z% is the photo‐stationary state distribution of *Z* isomer.

The bulkiness and electronic properties of halogen substituents affect AB ring coplanarity and, consequently, photochemical properties and thermal half‐lives of the *Z* isomer. For halogens, the pronounced differences in planarity primarily arise from steric hindrance, making atomic size the dominant factor influencing the geometry. As one progresses down in group 17 of the periodic table, the n–π* transition of the (*E*)‐TOAB isomer undergoes a bathochromic shift. This shift is primarily due to increased destabilizing interactions between the bulky halogen substituents and the lone pairs of the N═N. Larger halogens possess greater atomic radii, intensifying steric and electronic interactions with the adjacent nitrogen lone pairs. These interactions raise the energy level of the n MO, thereby reducing the energy gap between the n and π* orbitals. Consequently, this reduction leads to absorption of light of lower energy, resulting in a more pronounced bathochromic shift in the n–π* absorption band, as illustrated in Figure 8.^[^ ^50^ ^]^

The larger the halogen substituent, the more pronounced the red‐shift of the n–π* absorption band of the *E* isomer, which enables utilization of longer wavelengths for photoisomerization, ranging from 530 nm for fluorine to 660 nm for iodine. Conversely, PSD values exhibit the opposite trend: as the halogen size increases, there is less separation between the n–π* absorption bands of the *E* and *Z* isomers, resulting in lower percentage of *Z* isomer at PSS. The half‐life of the *Z* isomers decreases as the halogen size increases, following a pattern consistent with electronegative substituents relieving the N═N lone pair repulsion and stabilizing the overall energy of the *Z*‐isomer. A comprehensive comparison of all the properties is presented in Table 2.

**Table 2:** Comparison of the properties of tetra‐*ortho*‐halogen substituted ABs.^[^ ^50^ ^]^

*Ortho* substituent	*Para* substituent	λ_max_ (nm) n→π*	λ_excitation_ (nm)^b)^	PSD *Z* %	Half‐life^a)^ *t*_1/2_
F	NHBoc	454	530	84	1.25 years
Cl	NHBoc	463	625	77	160 h
Br	NHBoc	466	625	66	12 h
I	NHBoc	514	660	−	5 min
I	NPhth	512	660	43	3 min

^a)^ Half‐lives for the metastable *Z* isomers in the thermal *Z*‐to‐*E* isomerization process and photochemical properties measured in DMSO at 25 °C.

^b)^ Wavelength used for determination of photo‐stationary state distribution (PSD).

### 2.2 Chalcogens

**Figure 9:**
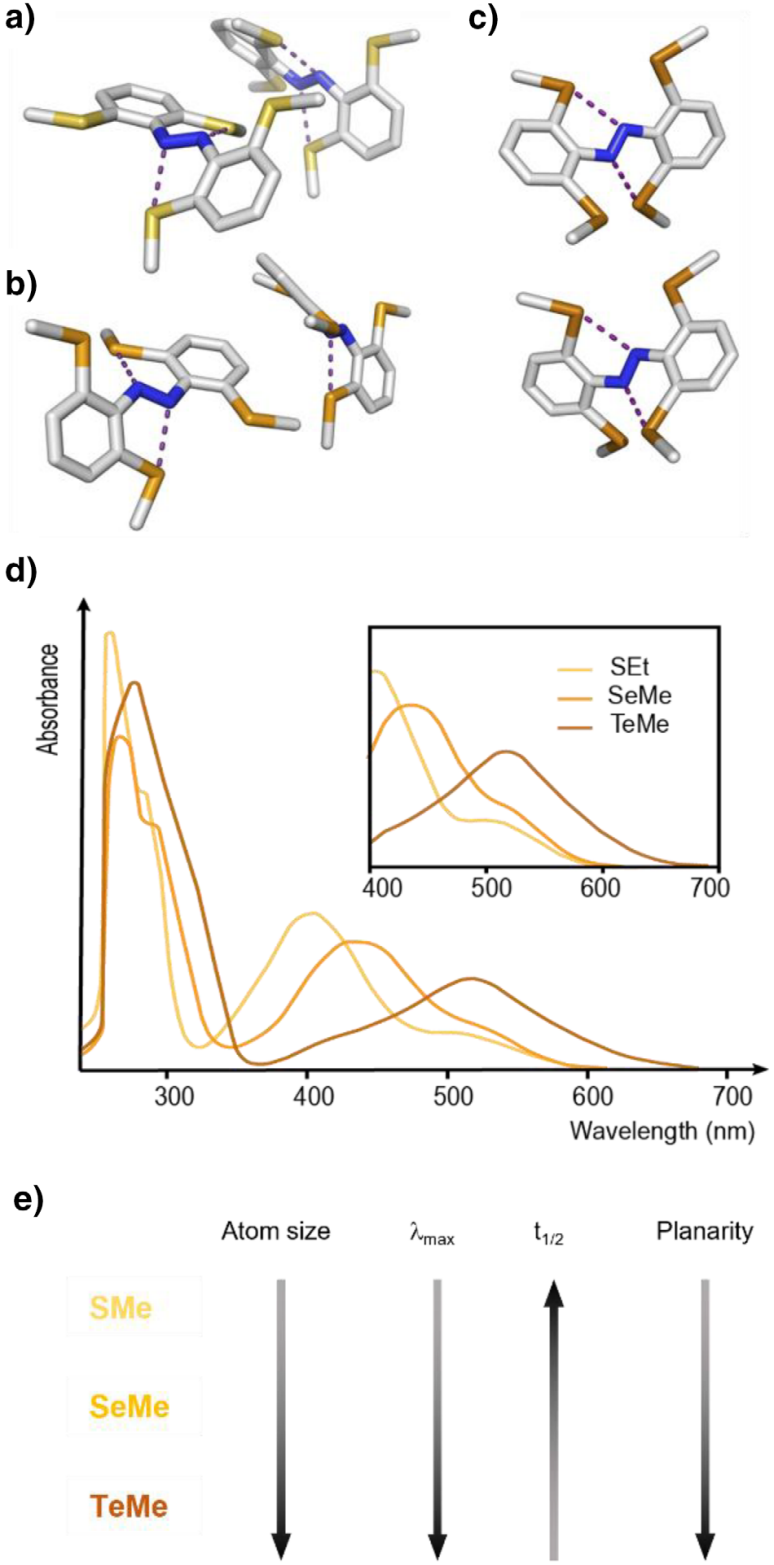
X‐ray crystal structure of (a) (MeS)_4_‐TOAB; b) (MeSe)_4_‐TOAB; c) (MeTe)_4_‐TOAB; d) Absorption spectra of tetra‐*ortho*‐chalcogen‐substituted TOABs. Adapted with permission from ref. [50] Copyright © 2022 The Royal Society of Chemistry. e) General trends exhibited by key (photo)chemical properties of tetra‐*ortho*‐ABs with chalcogen substitutions.

Similar to chlorine atoms in Cl_4_‐TOAB,^[^ ^37^ ^]^ X‐ray crystallography (Figure 9) reveals that the installation of *ortho*‐substituents in (MeS)_4_‐TOAB, (MeSe)_4_‐TOAB and (MeTe)_4_‐TOAB enables multiple conformations within the same unit cell, including more planar geometries of the *E* isomer.^[^ ^50^ ^]^ Interestingly, despite the increasing atomic size as one progresses down in the periodic table, the coplanarity of the aryl rings slightly increases. Unlike halogens, where the planarity is strongly influenced by atomic size due to steric hindrance, chalcogens can form intramolecular chalcogen bonds. These interactions exhibit an inherent preference for five‐membered intramolecular [S/Se/Te]···N interactions, leading to a less pronounced change in planarity compared to halogens.

Similar to the behavior observed with halogen substituents, larger groups at the *ortho*‐position result in more pronounced bathochromic shift of the n–π* transition band. This effect is attributed to the increased destabilizing interactions between the substituents and the lone pairs on the diazo nitrogen atoms. The combination of both increased instability of the n‐MO and the contributions of the minor (but present) populations of planar conformations results in a larger bathochromic shift of the n–π* bands as the atomic size of the chalcogen increases. The *E* isomer of SMe_4_‐TOAB displays a λ_max_ for the S_0_‐S_1_ transition at 514 nm, meanwhile the value for the same transition in (MeSe)_4_‐TOAB is further red‐shifted to 534 nm. Lastly, although for (MeTe)_4_‐TOAB this value does not show a pronounced bathochromic shift (λ_max_ = 519 nm), the absorption tail extends significantly compared to other chalcogens, exhibiting absorption beyond 600 nm, as shown in Figure 9.^[^ ^50^ ^]^

With respect to the photoswitching behavior, the chalcogen‐substituted TOABs display robust, forward switching using solely red light. Both (MeS)_4_‐TOAB and (MeSe)_4_‐TOAB can be switched from *E* to *Z* isomer by using 625 nm light. However, no photoisomerization was observed for (MeTe)_4_‐TOAB, probably due to strong chalcogen bonds formation in the *E* isomer between the tellurium and the azo group.^[^ ^55^ ^]^ Reverse isomerization occurs thermally within the seconds to minutes range. The decrease in half‐lives with substituent size increase is consistent with electronegative substituents mitigating repulsion between the N═N lone pairs, thereby stabilizing the energy of the *Z*‐isomer. (MeS)_4_‐TOAB exhibits a half‐life of 5 min, while (MeSe)_4_‐TOAB shows a half‐life of 80 s.^[^ ^50^ ^]^

Regarding the *para* substitution in this class of TOABs, π‐EWGs induce a red‐shift in the λ_max_ of approximately 80 nm in respect to the *para*‐unsubstituted compound. This enables the use of 660 nm light for the switching. Simultaneously, it also has an influence on the half‐life, decreasing its duration from minutes [for *para* unsubstituted (MeS)_4_‐TOAB and (MeSe)_4_‐TOAB] to seconds.^[^ ^50^ ^]^

### 2.3 Tetra‐*ortho*‐Methoxy‐Azobenzenes

The absorption spectrum of the *E* isomer of (MeO)_4_‐TOAB exhibits two separated bands. The first band, corresponding to the π–π* transition, has a maximum wavelength (λ_max_) of approximately 305 nm. The observed slight blue shift in the π–π* absorption relative to the parent unsubstituted azobenzene (−11 nm) indicates that the steric bulk of the *ortho‐*methoxy groups induces a twist in the two aryl rings of the *E* isomer, causing them to deviate from planarity.^[^ ^56^ ^]^ The second band, corresponding to the n–π* transition, has a λ_max_ of approximately 470–480 nm.^[^ ^48^ ^]^ The *E* isomer exhibits a significant bathochromic shift of +31 nm in the n–π* band when compared to the parent azobenzene. This phenomenon can be explained by the repulsion between the lone pairs of electrons on the nitrogen atoms and the oxygen atoms of the methoxy groups. This repulsion increases the energy level of the n‐MO (Figure 4d), thereby inducing a bathochromic shift. Conversely, the n–π* band of the *Z* isomer is similar in energy to that of the parent compound, with a λ_max_ of approximately 430 nm. In this instance, the notable ∼40 nm band separation results from the bathochromic shift of the *E* isomer. This characteristic enables high PSS ratios, achieving 83% of the *Z* isomer upon excitation at a wavelength of 530 nm and 67% of the *E* isomer upon excitation at 365 nm. The *Z* isomer is still relatively stable (> 16 h at 37 °C in DMSO), although less so than in the F_4_‐TOAB or Cl_4_‐TOAB derivatives.^[^ ^48^ ^]^

The effect of *para* substitution in (MeO)_4_‐TOAB is of particular interest. Substitution at this position with EWG, such as F, Cl or COOEt, as well as EDG groups like methyl,^[^ ^57^ ^]^ methoxy^[^ ^48^ ^]^ or ‐NHCOMe^[^ ^56^ ^]^ results in band separation comparable to that of the *para* unsubstituted MeO_4_‐TOAB (approximately 40 nm). However, in the case of the strong EWG such as NO_2_,^[^ ^57^ ^]^ band separation is notably reduced. In relation to their photoswitching behavior, irradiation of the differently *para*‐substituted (MeO)_4_‐TOABs mentioned above with light of 530–560 nm^[^ ^48^, ^56^ ^]^ enables obtaining PSDs consisting of 77%–83% of *Z* isomer. The reverse transition can be achieved using light of either 365 nm or 450–460 nm. Nevertheless, the use of blue light^[^ ^56^ ^]^ results in PSS with a higher content of the *E* isomer (85% *E*) compared to the use of UV light^[^ ^48^ ^]^ (54%–67% *E*). With respect to the half‐lives in DMSO, EDGs at the *para* position^[^ ^48^, ^56^ ^]^ significantly extend the half‐life (> 48 h) compared to that of the unsubstituted compound^[^ ^48^ ^]^ (> 16 h).

Finally, (MeO)_4_‐TOABs are of substantial interest amongst other TOAB because they allow for the formation of azonium ions at physiological pH in addition to enabling to tune the basicity of the azo bond by the introduction of different substituents on the *para*‐position.^[^ ^44^, ^58^ ^]^

### 2.4 Azonium Ions

Protonation of the azo bond in conventional ABs leads to the formation of azonium ions, which exhibit strong absorption in the far‐red/NIR region.^[^ ^40^, ^59^ ^]^ Despite these favorable photochemical properties, these unsubstituted azonium ions present two main drawbacks that prevent them from being considered as optimal chemical tools. First, most azonium ions are only formed in very acidic environments, presenting pK_a_ values in a range between −2.48 and −0.93^[^ ^60^ ^]^ Second, their *Z* isomers typically present half‐lives in the µs range, preventing their efficient accumulation under light irradiation.^[^ ^38^ ^]^

**Figure 10:**
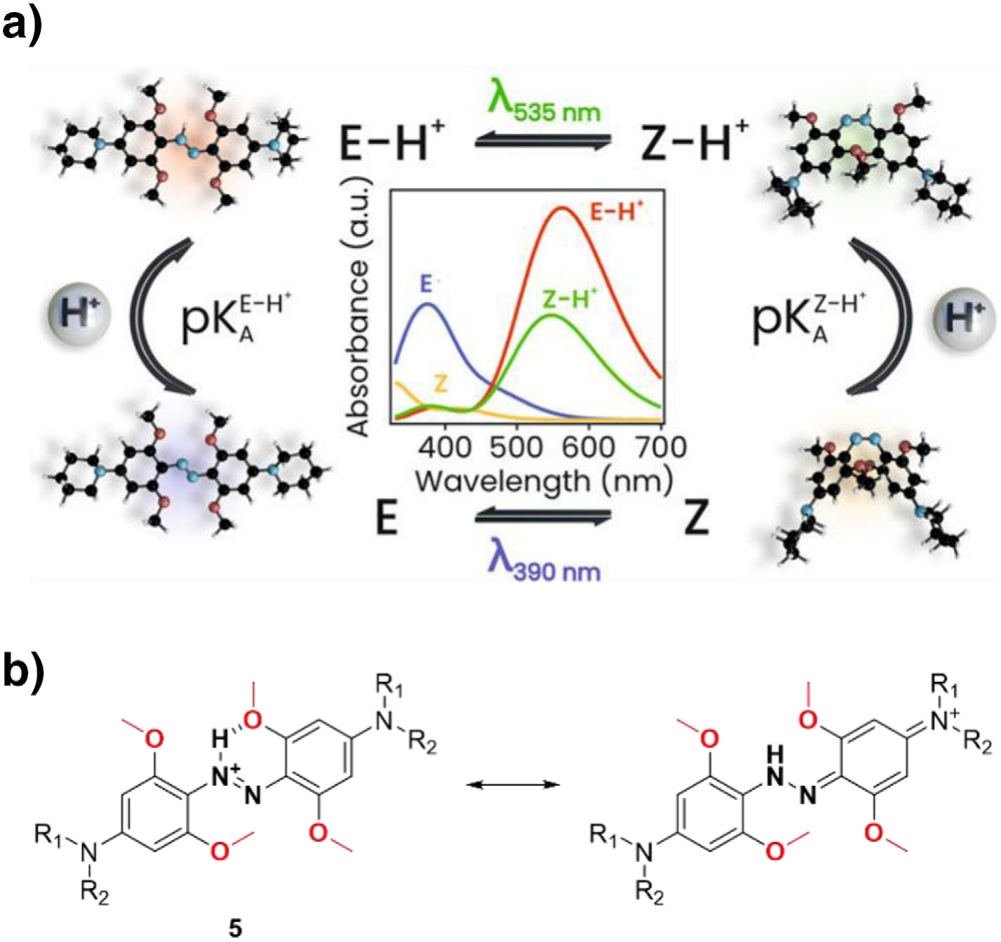
a) Equilibria between the photo‐isomers of (MeO)_4_‐TOABs and conjugated azonium ions and theoretical UV–Vis spectra of all four species involved; Adapted with permission from ref. [61] Copyright © 2023 American Chemical Society. b) Resonance structures of *para*‐amino substituted azonium ions.

In this context, (MeO)_4_‐TOABs are especially relevant because they allow for the formation of azonium ions at physiologically relevant pH levels. This phenomenon can be attributed to the formation of a hydrogen bond between the nitrogen atom of the azonium group and the oxygen atom of the methoxy group, an interaction that increases the pK_a_ of the azonium ion.^[^ ^38^ ^]^ Protonation of (*E*)‐(MeO)_4_‐TOAB disrupts the symmetry of the azo group, altering the electron density distribution of the TOAB and resulting in a quasi‐planar *E‐*H⁺ structure. This modification induces a bathochromic shift in the π–π* band, while n–π* transitions are no longer feasible due to the involvement of the nitrogen's lone electron pairs in protonation.^[^ ^61^ ^]^ Isomerization to the metastable isomer leads to a partial (pH dependent) deprotonation to the neutral *Z* species due to the lowered pK_a_ of the *Z*‐H^+^ species. This effect on pK_a_ can be explained by the fact that in the *Z* configuration, the methoxy groups are positioned far from the proton of the azonium ion, which prevents the formation of hydrogen bonds (Figure 10a).^[^ ^38^, ^61^ ^]^

The overall thermal isomerization process to the stable species of the system is highly pH‐dependent. The thermal relaxation of the azonium *Z* isomer (*Z‐*H⁺) occurs at a significantly faster rate compared to the one of the neutral species because of the greater single‐bond character of the N═N bond due to the presence of relevant resonance structures, as depicted in Figure 10b. However, this fast thermal relaxation is decreased because of the deprotonation of the *Z‐*H⁺ to the longer‐lived *Z* isomer. This allows for tunable half‐lives in the millisecond (at lower pH values) to second (at higher pH values) range, which are suitable for various applications.^[^ ^38^, ^61^ ^]^

The basicity of the azo bond is a key parameter for optimization, and it can be tuned by the substitution of the *para*‐position. (MeO)_4_‐TOAB shows the formation of the corresponding azonium ion at pH 2 and *para*‐methoxy‐(MeO)_4_‐TOAB at pH 4,^[^ ^48^ ^]^ similarly to *para*‐diacetamido‐(MeO)_4_‐TOAB.^[^ ^56^ ^]^ With stronger EDG, such as amines, the formation of the azonium ion can be achieved at physiological pH.^[^ ^38^ ^]^ Specifically, amines resembling pyrrolidine form azonium ions at neutral pH (with pK_a_ = 8.9), demonstrating photoswitching upon irradiation with 660 nm light and exhibiting a thermal lifetimes of ∼100 ms.^[^ ^59^ ^]^ Substitution at both *ortho* and *meta* positions by incorporating a fused dioxane ring and a methoxy substituent has enabled the synthesis of azonium ions with the most red‐shifted absorbance observed in this class of molecules to date, being able to isomerize under irradiation with 720 nm.^[^ ^62^ ^]^

## 3 Synthesis and Reactivity of TOABs

The synthesis of ABs has been thoroughly investigated over the last century due to the ever‐growing interest in their applications. Several methods are nowadays recognized as historically established strategies.^[^ ^63^ ^]^ Among them, the Baeyer‐Mills,^[^ ^64^ ^]^ the azo‐coupling^[^ ^65^ ^]^ and the oxidative coupling of anilines^[^ ^63^ ^]^ are regularly employed to generate a large variety of differently substituted ABs, including TOABs. However, the challenges associated with incorporating substituents in all *ortho* positions sometimes render those classical approaches inefficient due to the resulting steric hindrance and/or electronic effects of *ortho*‐substituents. Among all the TOABs, especially those bearing in their *ortho* position, alkoxy, alkylthio and iodine remain so far the hardest to synthesize. Therefore, in recent years, different approaches tailored specifically to the synthesis of TOABs have been investigated, including *ortho*‐lithiation^[^ ^37^, ^48^ ^]^ and late‐stage functionalization (e.g., inserting substituents in *ortho*‐positions of available ABs).^[^ ^57^, ^66^, ^67^, ^68^ ^]^ These methodologies aim to provide compatibility with a wide range of (often reactive) substituents in *para* positions, since *para‐*substituents can act as key handles to introduce additional functional groups, thereby enhancing versatility for various applications.

### 3.1 Synthetic Methods toward TOABs

#### 3.1.1 Classical Methods

##### Oxidative coupling/dimerization of anilines

**Scheme 1:**
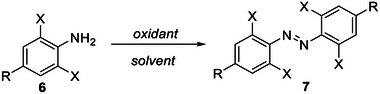
Typical oxidants and conditions used for synthesizing TOABs: 1) KMnO_4_/FeSO_4_·7H_2_O or CuSO_4_·5H_2_O in DCM, reflux or −78 °C; 2) AgO in anhydrous acetone, r.t.; 3) HgO, light (400 W) in MeCN, r.t.; 4) MnO_2_ in toluene, 130 °C; 5) CuBr/pyridine in toluene, 60 °C; 6) (*t*‐BuOO)_2_, 1 mol% [Cl_2_NN]Cu, benzene, 90 °C; 7) NaBO_3_.4H_2_O/Na_2_MoO_4_ in ACOH, 50 °C; 8) *t*‐BuOCl/NaI in *t*‐BuOH/Et_2_O, r.t.; 9) NCS/DBU in dichloromethane, −78 °C; 10) *i)* PIDA, TMSOTf *ii)* DMAP in DCM, r.t.; 11) PIDA/Ca(ClO)_2_, solvent free; 12) Pt electrode, NaNO_3_, pyridine in DMF.

The oxidative coupling of anilines (**6**, Scheme 1) has enabled the synthesis of TOABs bearing all the reported *ortho* substitutions patterns. Several mechanisms have been proposed for this transformation: when the oxidation is photochemically driven, it relies on the formation of cation radical on the aromatic amine, which undergoes a N–N coupling to yield an intermediate hydrazobenzene that is finally oxidized to the target TOAB **7**.^[^ ^63^ ^]^ Under other oxidation conditions,^[^ ^69^, ^70^, ^71^ ^]^ either halo‐intermediates or partially oxidized aniline^[^ ^67^ ^]^ are formed that finally react with remaining anilines yielding TOABs. The oxidative coupling/dimerization of anilines is particularly suitable (but not limited) to obtaining symmetric ABs,^[^ ^35^, ^41^ ^]^ and it usually tolerates EWG or alkyl substituents in *para* positions. Moreover, soon it could be possible to extend aniline cross‐dimerization^[^ ^69^ ^]^ to prepare non‐symmetric TOABs. The different oxidizing conditions explored for TOABs are herein discussed depending on the type of oxidants used and the related conditions.

The typically used oxidative coupling conditions are plagued by long reaction times and moderate yields. They include 1) KMnO_4_ combined with FeSO_4_·7H_2_O^[^ ^35^, ^36^, ^41^, ^50^ ^]^ or CuSO_4_·5H_2_O^[^ ^38^, ^44^ ^]^ in boiling dichloromethane, which is currently the most often tested approach for the majority of TOABs within the oxidative coupling methodology; 2) excess of AgO at room temperature or under reflux conditions with similar reaction times, which leads to yields around 10%^[^ ^44^, ^56^ ^]^; and 3) HgO under photo‐irradiation in acetonitrile at room temperature, which was employed for a limited number of TOABs allowing for mild and short reaction conditions^.[^ ^72^ ^]^ Less conventional conditions were tested for only few TOABs, and they include 4) MnO_2_ in toluene^[^ ^73^ ^]^ or 5) CuBr and pyridine in the same solvent^[^ ^50^, ^62^ ^]^; 6) di‐*t*‐butyl peroxide, 1 mol% [Cl_2_NN]Cu in benzene (this method is however not highly recommended considering the low yield obtained and the solvent toxicity);^[^ ^74^ ^]^ 7) NaBO_3_·4H_2_O and catalytic amounts of Na_2_MoO_4_ in glacial acetic acid at 50 °C for 1 h,^[^ ^75^ ^]^ which has resulted in a consistent alternative to conditions (6) for obtaining 1,2‐bis(2,4,6‐trichlorophenyl)diazene (90% vs. 5% and more feasible reaction conditions). Milder oxidation conditions also often prove sufficient to provide good to excellent yields of TOABs, and they include 8) metal‐free methods that use *t*‐BuOCl and NaI in *t*‐BuOH, in short reaction times at room temperature, affording high yields;^[^ ^69^ ^]^ 9) NCS (N‐Chlorosuccinimide) and DBU (1,8‐Diazabicyclo(5.4.0)undec‐7‐ene) in dichloromethane at −78 °C to room temperature, useful to provide a wide variety of TOABs in short reaction times;^[^ ^50^, ^70^ ^]^ 10) Weiss’ reagent using PIDA ((Diacetoxyiodo)benzene), TMSOTf and Dimethylaminopyridine (DMAP) in dichloromethane at mild conditions (room temperature) but in quite long reaction times (2 days);^[^ ^50^ ^]^ 11) oxidative‐mechanochemistry methods that paved the way to apply green chemistry in the synthesis of TOABs by using again PIDA (or Ca(ClO)_2_) in a grinding reactor;^[^ ^76^ ^]^ finally, 12) electrochemical methods were pioneered in the synthesis of TOABs by using pyridine and NaNO_3_ in presence of Pt electrodes.^[^ ^63^ ^]^ Recently, dinickel catalyst has been used for the synthesis of TOABs amongst others hindered ABs. This methodology relies on the dimerization of azide substrates allowing to obtain TOABs in moderate to excellent yields.^[^ ^58^ ^]^

##### Azo‐coupling reaction

**Scheme 2:**
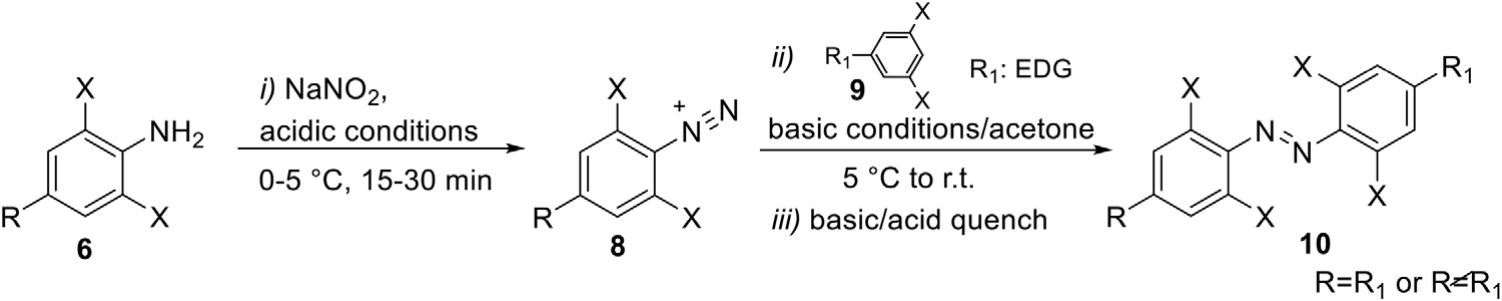
Typical azo‐coupling conditions explored for TOABs.

The azo‐coupling reaction relies on the formation of the diazonium salt **8**, a weakly electrophilic specie that is obtained by diazotisation of an aromatic amine **6** and easily reacts with electron‐rich aromatic compounds **9** (acting as nucleophiles) usually in their *para* position in respect to the EDG substituent.^[^ ^63^, ^65^ ^]^ The azo‐coupling strategy has been investigated for obtaining both symmetric and non‐symmetric TOABs **10**,^[^ ^44^, ^51^, ^77^, ^78^ ^]^ generally giving products in moderate yields. TOABs that can be accessed this way feature several EWG (NO_2_, Br) and EDG (OH, NH_2_/NRR_1_) substituents, where the latter play a key role in enhancing the electron‐rich character of the aromatic species **9** reacting with the in situ formed diazonium salt **8**. Despite the modest yield, the method is worthy of further exploration, providing easy access to multiple non‐symmetric TOABs with good functional group tolerance (Scheme 2).

##### Baeyer‐Mills reaction

**Scheme 3:**
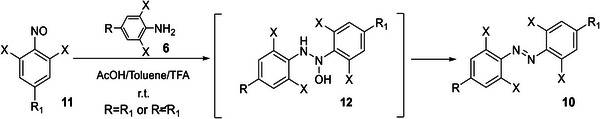
Baeyer‐Mills conditions explored for TOABs.

The Baeyer‐Mills method involves a straightforward condensation reaction between a nitroso derivative **11** and an aromatic amine **6**, typically under acidic conditions. This approach is frequently applied with anilines presenting EDG‐*para* substituents and *para*‐EWG nitroso compounds for synthesizing non‐symmetric ABs. Specifically for TOABs, it has been investigated for both symmetric and non‐symmetric candidates (R ≠ R_1_, compound **10**, Scheme 3).^[^ ^35^, ^46^ ^]^ The nitroso intermediates are usually prepared using a two‐ or three‐fold excess of potassium peroxymonosulfate (e.g., Oxone) in biphasic media, obtaining excellent yields (over 95% in the latter case).^[^ ^35^, ^46^ ^]^

#### 3.1.2 Emerging Methodologies for the Preparation of TOABs

Although some of the traditional methodologies discussed above work efficiently for the synthesis of many TOABs, none of them has provided a general synthetic strategy that can be routinely applied for every TOAB‐bearing synthetic target. This has driven the development of new methodologies tailored for TOABs synthesis in the last decade, expanding the repertoire of available methods.

##### *Ortho*‐lithiation

**Scheme 4:**
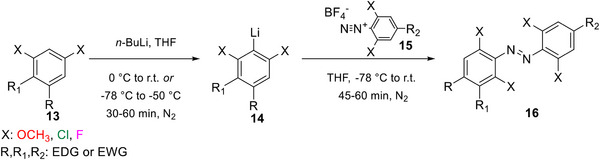
*Ortho*‐lithiation conditions explored for TOABs.

This method (Scheme 4) employs initial directed *ortho*‐lithiation of 1,3‐disubstituted benzenes (X = OMe, Cl, F, compound **13**, Scheme 4) to access 2,6‐disubstituted lithium species **14** that then react as nucleophiles with aryldiazonium counterparts **15** to provide symmetric and non‐symmetric TOABs **16**.^[^ ^37^, ^48^ ^]^

The substrate scope of this methodology highlights its versatility, as it tolerates *para* EWG, EDG and alkyl substituents, covering the Hammet σ‐*para* constants range.^[^ ^37^, ^48^, ^79^ ^]^ Still, the lithiation conditions represent a significant synthetic drawback, together with limited stability and synthetic availability of aromatic compounds that can act as 2,6‐disubstituted lithium species or aryldiazoniums. To overcome these issues, the use of organolithium gels^[^ ^80^ ^]^ is a potential strategy that could significantly improve both the reaction's handling and safety.

##### Late‐stage functionalization approaches

**Scheme 5:**
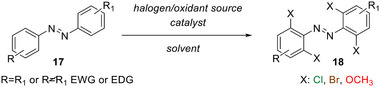
Typical late‐stage reaction conditions explored for TOABs: 1) *X = Cl*: NCS, Pd(OAc)_2_, acetic acid, 140 °C, o.n. or MW irradiation 1 h; 2) K_2_S_2_O_8_, Pd(PPh_3_)_4_, DBDMH (*X = Br*) or TCCA (*X = Cl*),1,2‐dichloroethane, 110 °C, 2 h; 3) *X = OMe*: Pd(OAc)_2_, PIDA, toluene/alcohol or diol, 40 °C, 16 h; 4) *i) X = Br*: K_2_S_2_O_8_, Pd(PPh_3_)_4_, DBDMH, Cu(OTf)_2_‐H_2_O, 1,2‐dichloroethane, 110 °C, 2 h; *ii) X = OMe*: CuCl, NaOMe, HCOOMe, MeOH, 115 °C, 16 h.

The late‐stage functionalization approaches (Scheme 5) offer access to both symmetric and non‐symmetric TOABs, with a very good tolerance for many EWG substituents in *para* or *meta* position to the diazo bond. Direct, Pd‐mediated C‐H activation has been tested on AB **17** for the straightforward synthesis of several TOABs **18** (Cl, Br, OMe/OR) with the sole exception of F_4_‐TOABs.^[^ ^57^, ^66^, ^67^, ^68^ ^]^

The late‐stage chlorination approach for TOABs has been recently studied by Trauner and colleagues^[^ ^67^ ^]^ who optimized the C‐H halogenation reaction to obtain Cl_4_‐TOABs, avoiding the negative interference of steric bulk around the N═N with the Pd coordination. The methodology is suitable for both symmetric and non‐symmetric ABs, accepting a wide range of *para*‐EWG groups; however, EDG groups, such as amines, directly inserted in *para* positions are not tolerated, while in the presence of alkyl groups terminated with OH or NH_2_ groups, the reaction provides *O*‐ and *N*‐acylated products. Despite the applicational limit in case of *para*‐EDG, the late‐stage chlorination conditions have been optimized under microwave irradiation, significantly shortening the reaction time and pioneering a green chemistry approach in the late‐stage TOABs synthesis.^[^ ^81^ ^]^ In 2019, Liu and colleagues^[^ ^68^ ^]^ have implemented late‐stage strategies for Br_4_‐TOABs and Cl_4_‐TOABs, investigating the detailed reaction mechanism that involves radical processes. This methodology provides an extended substrate scope and shorter reaction times and substituents positions in respect to Trauner's work.^[^ ^67^ ^]^

The most recent investigations of late‐stage functionalization aimed at extending the synthetic possibilities for (MeO)_4_‐TOABs.^[^ ^57^, ^66^ ^]^ Thorn‐Seshold and colleagues studied a versatile Pd‐catalyzed strategy for *ortho*‐alkoxylation of AB.^[^ ^57^ ^]^ The *ortho*‐substitution pattern has been evaluated for different, mixed or *ortho*‐OR moieties, including OEt, OMe, OPr, O(CH_2_)_3_OH to tune the overall hydrophilic‐hydrophobic‐water solubility character or to introduce isotopic labeling (OCD_3_) of final TOABs. Finally, Just‐Baringo and co‐workers^[^ ^66^ ^]^ aimed at widening the substrate scope for the synthesis of (MeO)_4_‐TOABs in a two‐step procedure. Their methodology starts by a reported synthesis of Br_4_‐TOABs,^[^ ^70^ ^]^ which is followed by substituting bromines for methoxy groups, providing differently substituted (MeO)_4_‐TOABs.However, despite a wide substrate scope, the methodology remains ineffective for synthesizing TOABs with amino‐substituents in *para*‐position that are valuable azonium ion precursors, limiting the overall methodology's versatility.

### 3.2 Preferred Synthetic Methods Related to *Ortho*‐substitution Patterns

**Figure 11:**
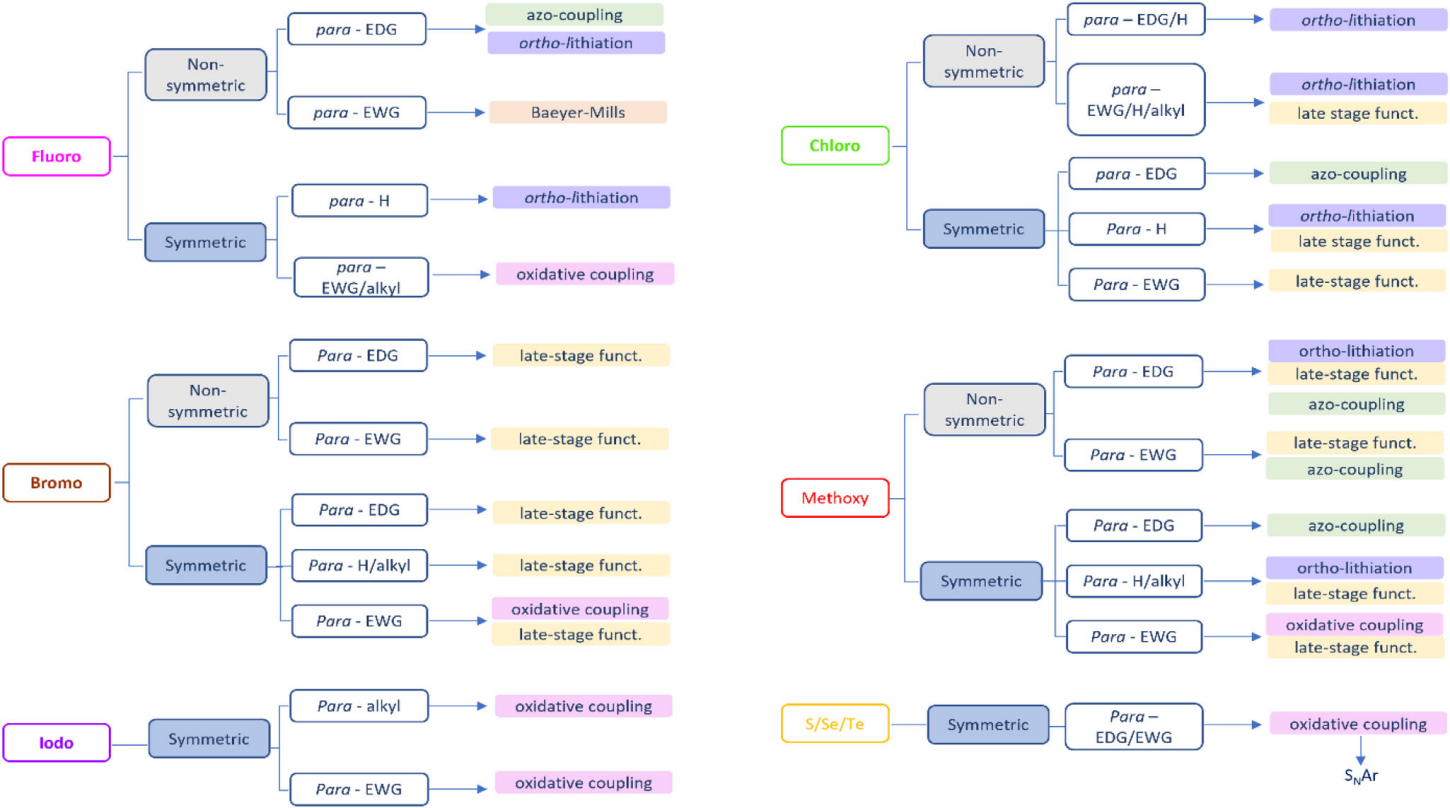
Decision tree on the preferred synthetic methodologies for TOABs.

With this section, we aim to summarize the information given above with a simple decision tree (Figure 11) for selecting the best synthetic strategies available for obtaining specific TOABs according to the *ortho*‐position and the overall substitution patterns.

F_4_‐TOABs have been obtained through almost all the synthetic strategies reported above, which highlights the Baeyer‐Mills method as a good option for synthesizing non‐symmetric F_4_‐TOAB with *para‐*EWG‐substituents,^[^ ^35^, ^46^ ^]^ while the *ortho*‐lithiation^[^ ^39^, ^48^, ^82^ ^]^ and azo‐coupling^[^ ^77^, ^83^ ^]^ result in valuable options for obtaining non‐symmetric candidates, including ones with mixed *para*‐EDG and EWG. The mild oxidative coupling^[^ ^50^, ^69^, ^70^ ^]^ remains instead a first‐choice strategy for symmetric candidates.

A similar situation is observed for the synthesis of Cl_4_‐TOAB, where also late‐stage approaches have been investigated. Several non‐symmetric compounds have been synthesized by *ortho*‐lithiation^[^ ^37^, ^48^ ^]^ and the late‐stage functionalization^[^ ^67^, ^68^, ^81^ ^]^ with diversified *para*‐H/alkyl, H/EWG or EWG substituents. Symmetric Cl_4_‐TOAB featuring *para*‐EDG substituents (e.g., NH_2_) can be obtained in moderate yield by azo‐coupling followed by reduction.^[^ ^44^, ^51^ ^]^ The exploration of Baeyer‐Mills methodology for non‐symmetric Cl_4_‐TOAB remains a synthetic opportunity worthy of evaluation, considering the good results obtained with F_4_‐TOAB. The synthesis of Br_4_‐TOABs and I_4_‐TOABs has been approached by both oxidative coupling^[^ ^44^, ^50^, ^75^ ^]^ and late‐stage functionalization^[^ ^62^, ^64^ ^]^ giving easy access to both symmetric and non‐symmetric TOABs.

(MeO)_4_‐TOABs are of particular interest as precursors of azonium ions, especially with substitution patterns that increase the pK_a_ of the protonated form (i.e., strongly electron‐donating amine groups). In their synthesis, particularly the *ortho*‐lithiation^[^ ^48^ ^]^ and the late‐stage functionalization^[^ ^57^, ^66^ ^]^ have proven successful in building huge libraries of either symmetric and non‐symmetric candidates, showing however limitations for EDG‐substituents.^[^ ^48^, ^66^ ^]^ The synthesis of less conventional (EtS)_4_‐TOABs has been initially approached by oxidative coupling^[^ ^36^ ^]^ showing however low yield due to competitive oxidations and significant steric hindrance. To overcome these synthetic drawbacks, the S_N_Ar reaction on pre‐synthesized F4‐TOABs has been successfully employed^[^ ^50^ ^]^ extending the methodology to (MeS)_4_‐TOABs, (*i*PrS)_4_‐TOABs and other chalcogens, such as selenium and tellurium.

The five synthetic methods available for obtaining TOABs still remain insufficient for providing access to a class of molecules of such high application potential. The steric hindrance and the electronic effect on the reactivity of *ortho*‐substituents result in difficulties in establishing straightforward synthetic strategies. Furthermore, the application of green chemistry methodologies is still limited for TOABs^[^ ^76^, ^81^ ^]^ while being investigated much more for classical ABs (mechanochemistry,^[^ ^84^ ^]^ MW^[^ ^85^ ^]^ and flow‐chemistry^[^ ^86^ ^]^). New synthetic efforts should focus on the development of more sustainable approaches for TOABs, and the methodology development should also be directed toward a universal synthetic approach for TOABs.

### 3.3 Reactivity and Stability of TOABs

This section outlines the typical approaches taken in functionalizing TOABs for their applications. In this context, one needs to keep in mind that the presence of tetra‐*ortho* substituents on the TOAB scaffold significantly impacts the reactivity of the AB core, which can both be seen as an advantage in functionalization and as a cause of possibly impaired stability under the application condition.

**Scheme 6:**
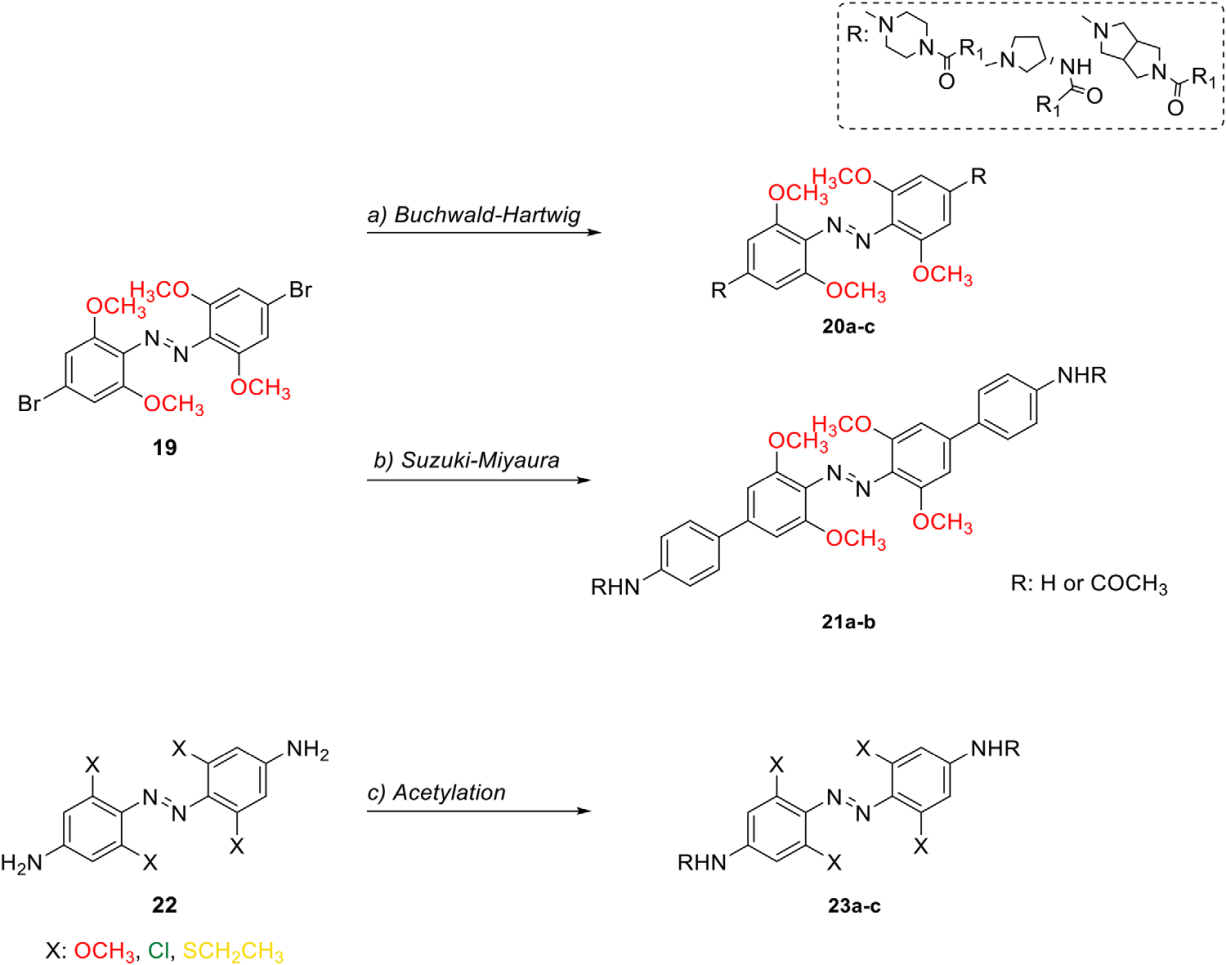
Functionalization approaches for TOABs. Typical reaction conditions: a) amine, Pd_2_(dba)_3_, Cs_2_CO_3_, RuPhos, toluene, 100 °C, N_2_; b) phenylboronic acid, Pd(PPh_3_)_4_, NaHCO_3_/K_2_CO_3_, anhydrous 1,2‐dimethoxyethane/1,4‐dioxane, 90/110 °C; c) acetyl chloride, pyridine/Et_2_O or triethylamine/CHCl_3_.

Halogen moieties located in *para*‐position of TOABs have been extensively used for introducing structural modifications. *Para*‐bromide group in (MeO)_4_‐TOAB **19** was reacted under Buchwald‐Hartwig‐like conditions (Scheme 6a) with amino‐substituents in satisfactory yields (over 80%) to provide libraries of azonium precursors **20a‐c**.^[^ ^59^, ^62^ ^]^ The Suzuki‐Miyaura coupling (Scheme 6b) involving **19** and aryl‐boronic moieties results in a successful approach to obtain TOABs with extended conjugation **21a‐b** in moderate yields (33%–54%); however, further evaluation of solvents and reaction conditions could be useful in improving the success of this coupling reaction.^[^ ^44^ ^]^ The Suzuki‐Miyaura coupling has been also successfully used in a three‐components‐one‐pot approach within the *ortho* lithiation methodology to successfully extend the libraries of available TOABs with minimal purification requirements.^[^ ^48^ ^]^ (MeO)_4_‐TOABs, Cl_4_‐TOABs, (EtS)_4_‐TOABs bearing *para*‐NH_2_ **22** easily undergo acetylation reactions (Scheme 6c), extending TOABs’ functionalization possibilities within the *para* positions **23a‐c** with a good yield (over 60%).^[^ ^36^, ^44^ ^]^

**Scheme 7:**
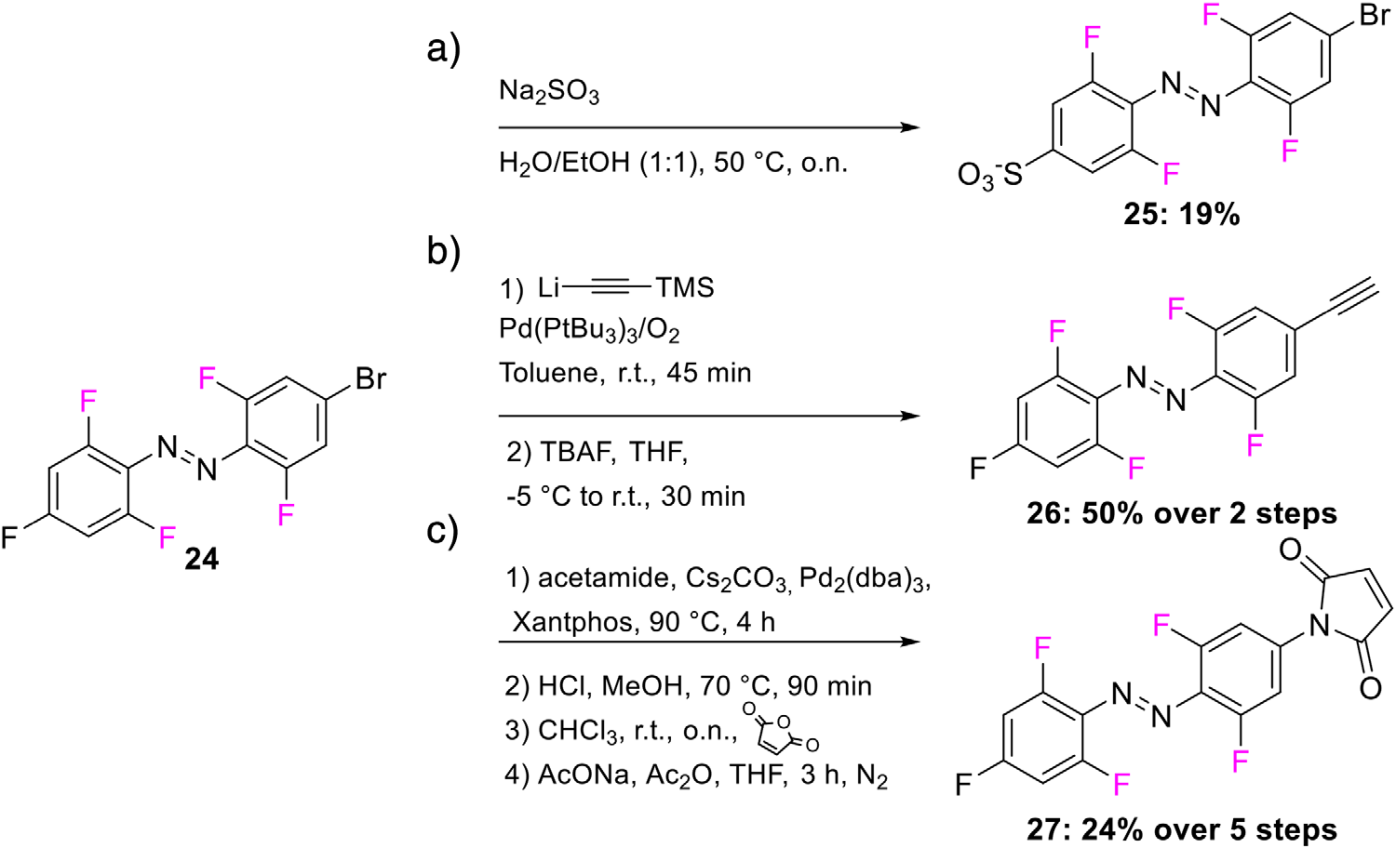
Orthogonal post‐functionalization possibilities explored for F_4_‐TOABs such as a) introduction of solubilizing moieties and introduction of b) alkyne group or c) maleimide moiety suitable for further click chemistry reactions.

The introduction of *para*‐substituents in F_4_‐TOABs deserves particular attention, thanks to the high electronegativity of the fluorine atom, which enables the S_N_Ar approach to TOAB functionalization. Especially the possibility of synthesizing non‐symmetric candidates with mixed Br or F substituents in the *para* positions **24** has paved the way for the next generation of orthogonal ABs’ functionalizations (Scheme 7).^[^ ^46^ ^]^ S_N_Ar can be used in F_4_‐TOABs in an orthogonal approach with respect to cross‐couplings, opening synthetic possibilities for differently bi‐functionalized building blocks^[^ ^46^ ^]^ particularly oriented toward further synthetic applications of click chemistry directed toward chemical biology outcomes **25–27**.

The reactivity of TOABs is closely correlated with their stability, especially in biological context where both the reactivity with biomolecules and metabolic degradation (enzymatic, including oxidations and reductions) have to be taken into consideration.^[^ ^87^, ^88^, ^89^ ^]^

The stability of TOABs has been evaluated with a particular focus on the reducing role of the pseudotripeptide glutathione (GSH) in cellular environments.^[^ ^87^, ^90^ ^]^ In general, GSH reacts toward ABs in two steps: a nucleophilic attack of thiol on the azo bond to create an intermediate adduct, which then undergoes a second reaction with GSH providing the reduced AB form (hydrazo compound) and the oxidized GSH dimer.^[^ ^87^ ^]^ Evaluation of reductive stability on F_4_‐TOAB bearing water‐solubilizing moieties has shown significant stability in the presence of GSH in vitro.^[^ ^46^ ^]^ Azonium ions instead proved insufficiently resistant to reduction, especially for the *E* isomeric form in the presence of GSH,^[^ ^44^ ^]^ while Cl_4_‐TOABs bearing the same *para* substitution pattern ensure stability in the presence of GSH. The high pK_a_ of the azo group in the presence of *ortho*‐OMe groups, combined with the planar conformation of the molecule when protonated, probably favor nucleophilic attack by GSH.^[^ ^44^ ^]^ However, when the GSH tripeptides are synthetically linked to bicyclic pyrrolidines in both *para*‐positions of azoniums, the reduction rates are significantly slowed down without any significant thiol/GSH physiological interference.^[^ ^59^ ^]^ Lastly, the substitution of *ortho*‐OMe with *ortho*‐SEt results in higher stability to GSH reduction, probably thanks to a decrease in resonance delocalization and a weaker H‐bonding capability, providing candidates stable to 10 mM GSH and more suitable for intracellular applications in respect to the *ortho*‐OMe analogs.^[^ ^36^ ^]^ The same trend has been recently observed in a more complex comparison of all the tetra‐*ortho* substitutions patterns explored by now as F, Cl, Br, I, OMe, SMe, SeMe bearing methylamine as the substituent in *para* positions, in the presence of 10 mM GSH.^[^ ^50^ ^]^ This analysis highlights how the higher stability to reduction is observed for the bigger and less electron‐withdrawing substituents (SeMe, SMe) in comparison to the halogens, confirming how the steric hindrance and the capability of the azo bond polarization play a key role in overall stability.^[^ ^50^ ^]^

A more comprehensive evaluation on the metabolic stability of TOABs will have to be considered in the near future, especially considering applications in the biomedical context,^[^ ^91^ ^]^ analyzing among others the stability toward reductases that populate intestinal bacteria or liver microsomes.^[^ ^87^ ^]^ The oxidative metabolism should be similarly studied for impacting on the aromatic substituents that can be responsible for *N*‐dealkylation or hydroxylation phenomena, among others, while minimally impacting on the diazo bond.^[^ ^10^, ^88^, ^89^, ^91^ ^]^ The potential cancerogenic effects should be similarly considered: fully reduced TOABs generates aniline metabolites of potential high toxicity, while metabolites with maintained diazo bond could be responsible of forming toxic DNA‐adducts.^[^ ^89^ ^]^ The final overall evaluation should be addressed to understand, both in vitro but particularly in vivo, the complete stability and toxicity profile of TOABs in relation to potential delivery and patient‐like administrations modalities to strengthen further biomedical‐oriented applications.

## 4 Current Applications of TOABs

TOABs have been introduced in multiple functional molecular systems, thanks to their compact structure and the capability of being operated with visible light in both directions. The following section outlines key examples of diverse applications that emerged within the last decade, divided into two main categories: biomedical applications (chemical biology, photopharmacology) and material sciences (photocontrol of polymers, NPs, supramolecular constructs).

### 4.1 Biomedical Applications of TOABs

TOABs are the most often applied visible light responsive photoswitches for enabling photocontrol of bioprocess to tune function of biomolecules (proteins, nucleic acids, lipids) and bioactive compounds. Table 3 provides an overview of TOABs’ molecular structures employed, switching properties and final targets.

**Table 3:** Overview of TOABs applications in biomolecules and bioactive compounds.

Entry	Cpd.	Application	Structure	Switching Wavelengths^a)^	Ref.
*Nucleic acids*					
*1*	**28**	Netropsin/DNA intercalator	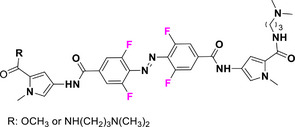	520/405 nm	[92]
*2*	**29**	G4‐binders	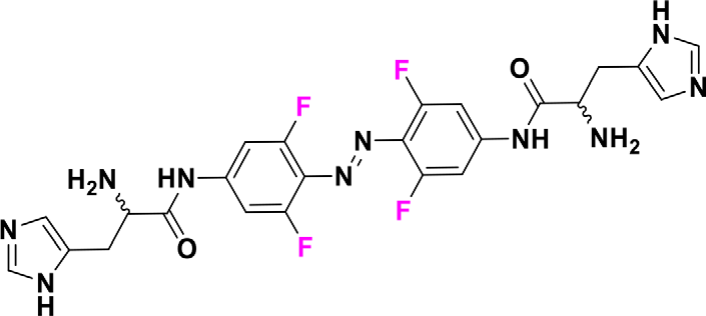	485–436 nm	[93]
*3*	**30**	siRNA	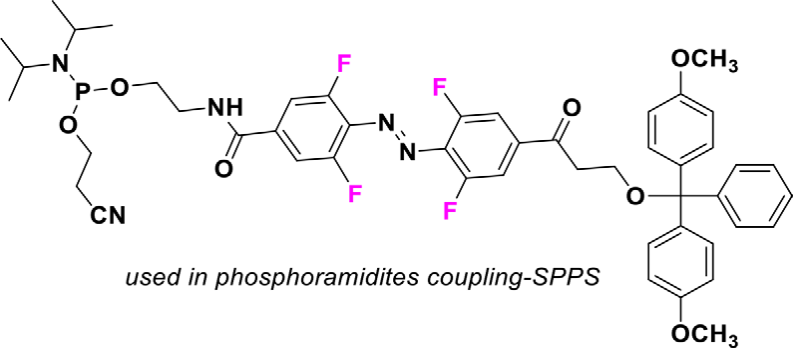	530/470 nm	[94]
*4*	**31**	Photo‐responsive PNA for DNA hybridization	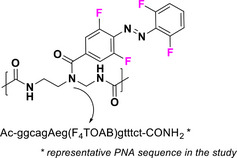	520/405 nm	[95]
*Peptides and proteins*					
*5*	**32**	Antibacterial activity (Tyrocidine A)	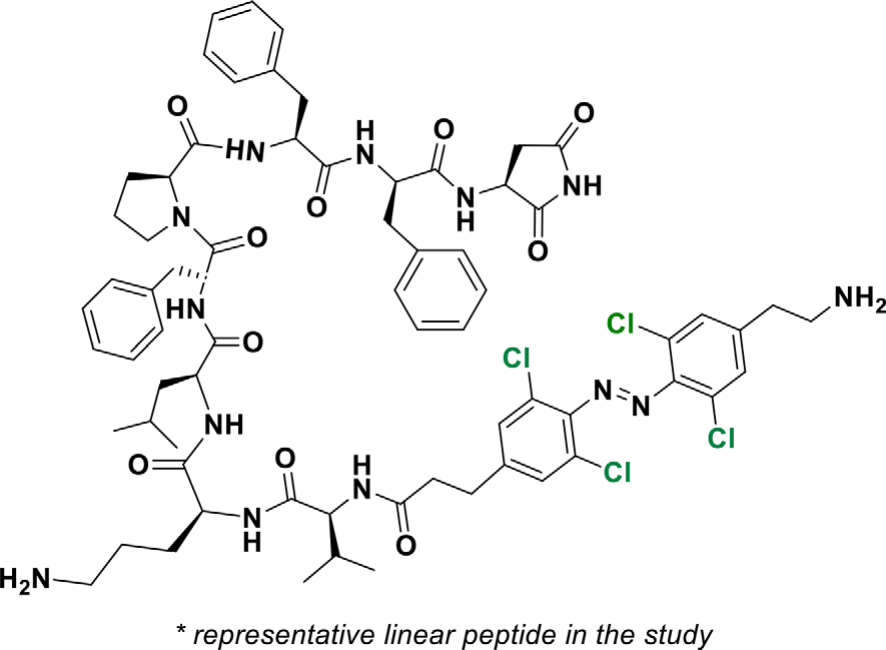	650 nm/daylight	[99]
*6*	**33**	CPP‐LK1	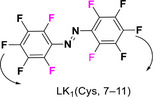	590/450 nm	[97]
*7*	**34**	PMI stapling	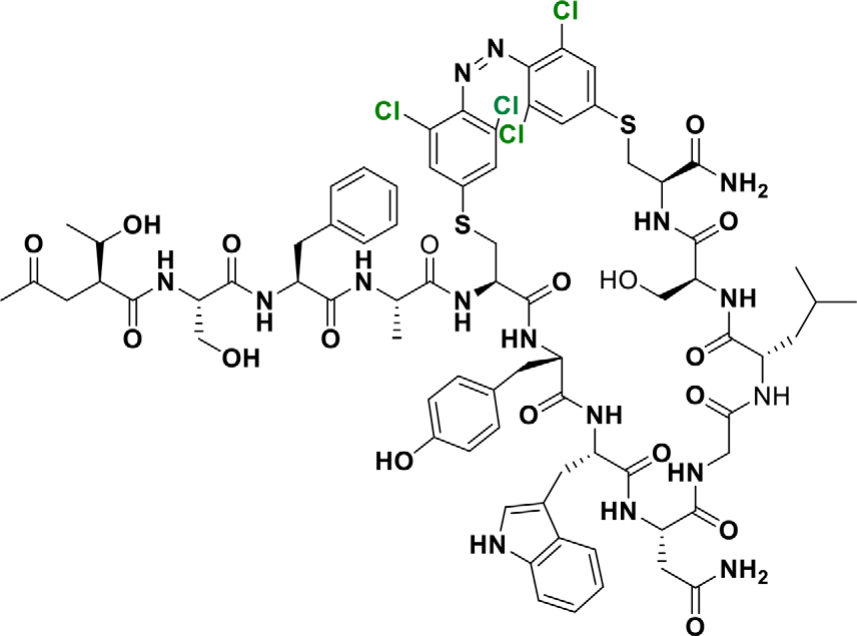	660/415 nm	[98]
*8*	**35**	Peptidomimetics MLL1 inhibitor	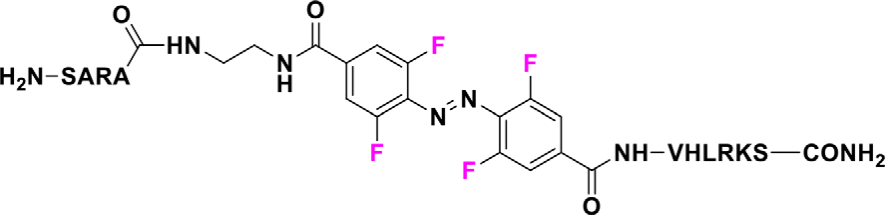	520/405 nm	[100]
*9*	**36**	Protein toxicity (FraC)	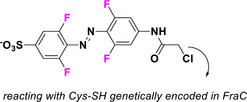	530/430 nm	[96]
*Membrane transport*					
*10*	**37**	Transmembrane anion transporter	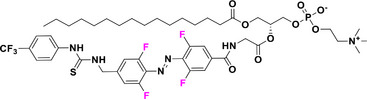	530/405 nm	[102]
*11*	**38**	Ion transporters	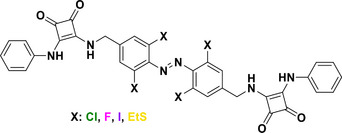	625/455 nm or thermal	[103, 104]
*Drug delivery*					
*12*	**39**	Drug delivery (Doxorubicin)	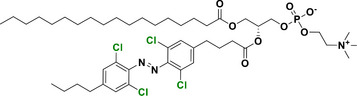	660 nm	[105]
*Small bioactive compounds*					
*13*	**40**	Antibacterial activity (Trimethoprim)	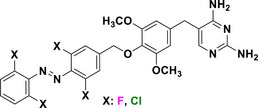	527(F)‐652(Cl)/ 400 nm	[106]
*14*	**41**	PROTACs	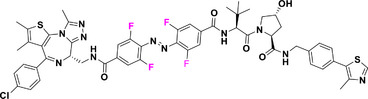	530/415 nm	[79]
*15*	**42**	Agonist of TRPV1	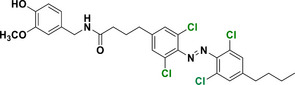	500–590/410–480 nm	[107]
*16*	**43**	Ion channel antagonist	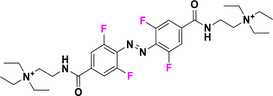	533/405 nm	[108]
*17*	**44**	Nicotinic acetylcholine receptor	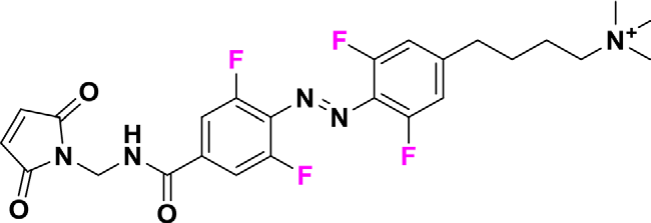	530/405 nm	[82]

^a)^ Switching wavelengths for *E* →*Z*/*Z* → *E*, respectively. Values given as range highlight two available wavelengths for switching.

#### 4.1.1 Regulation of Nucleic Acid Functions

TOABs have been used to enable light‐control over natural or synthetic nucleic acids through both non‐covalent interactions (binding, intercalation) between the target and TOAB‐modified targeted compounds and by the direct (covalent) incorporation into nucleic acids.

A few non‐covalent approaches have been recently studied using TOABs. Netropsin, a natural minor groove DNA binder, was modified by incorporation of F_4_‐TOABs through amide bonds to generate visible light photo‐responsive DNA‐targeted tools.^[^ ^92^ ^]^ Compound **28** (Table 3) showed efficient, reversible *E*/*Z* isomerization under irradiation with 520 and 405 nm light. Fluorescent intercalator displacement assays, supported by circular dichroism and UV‐Vis titration experiments, proved successful light‐controlled DNA‐binding affinity (in the nM range), with the *E* isomer being a stronger binder.

The targeting of G‐quadruplex (G4) has been approached by light‐controlled compounds to control their natural activity due to G4's role in the regulation of many biological processes. Compound **29** (Table 3)^[^ ^93^ ^]^ has been obtained by linking through Buchwald‐Hartwig reaction a F_4_‐TOAB functionalized with *para*‐Br substituents to D/L‐histidine residues. Their isomerization (*E* to *Z*) could be achieved with visible light (> 485 nm or 436 nm), and studies in light‐dependent thermal melting assays have shown that the *Z* isomer stabilizes both left and right‐handed G4 derivatives better when compared to the *E* isomer, which showed lower melting temperatures. Hence, the compounds have allowed to investigate G4 interactions by combining both chiral function and light‐induced properties for the final construction of chiroptical systems.

The covalent incorporation of TOAB within nucleotide targets has been recently explored by incorporating a F_4_‐TOAB (compound **30**, Table 3), though solid phase peptide synthesis (SPPS), into a short interfering RNA (siRNA), inspired by the growing interest in siRNAs as therapeutics.^[^ ^94^ ^]^ The synthesized photo‐responsive construct showed a maintained knock‐down activity (ON state) in the *E* form, while it was inactive (OFF state) in the *Z* isomer that is obtained upon irradiation with 530 nm light. The OFF state was maintained for up to 72 h without interfering with the switching capability, and irradiation with 470 nm light resulted in efficient restoration of the *E* isomer and the related activity. Further investigations should shed light on the precise activation/inactivation mechanism, highlighting high potentialities for the light‐controlled therapeutic use.

The light‐control of the pairing between artificial nucleotide analogs PNA (peptide nucleic acids) and complementary ssDNA has been evaluated by inserting F_4_‐TOABs into PNA sequences^[^ ^95^ ^]^ (compound **31**, Table 3). Different PNAs with F_4_‐TOABs incorporated by amide coupling were evaluated for their light responsivity: irradiation with 520 nm light was successful in obtaining the *Z* isomer of F_4_‐TOAB inserted in the sequences, while the back isomerization to the *E* one was achieved by irradiation with 405 nm, translating the light responsivity from F_4_‐TOAB to the whole PNA. The melting temperature analyses showed a negatively perturbed pairing capability in the presence of F_4_‐TOABs, but peculiar cases of sequences with two F_4_‐TOABs showcased a detectable difference in the pairing capability depending on the TOABs’ *Z* or *E* form showing promising future for their application in this context.

#### 4.1.2 Regulation of Peptides and Proteins Activity

The incorporation of TOABs within peptides and proteins has resulted in efficient control of peptides’ structure and conformation, as well as in the overall function and properties of complex proteins for many diversified applications.

**Figure 12:**
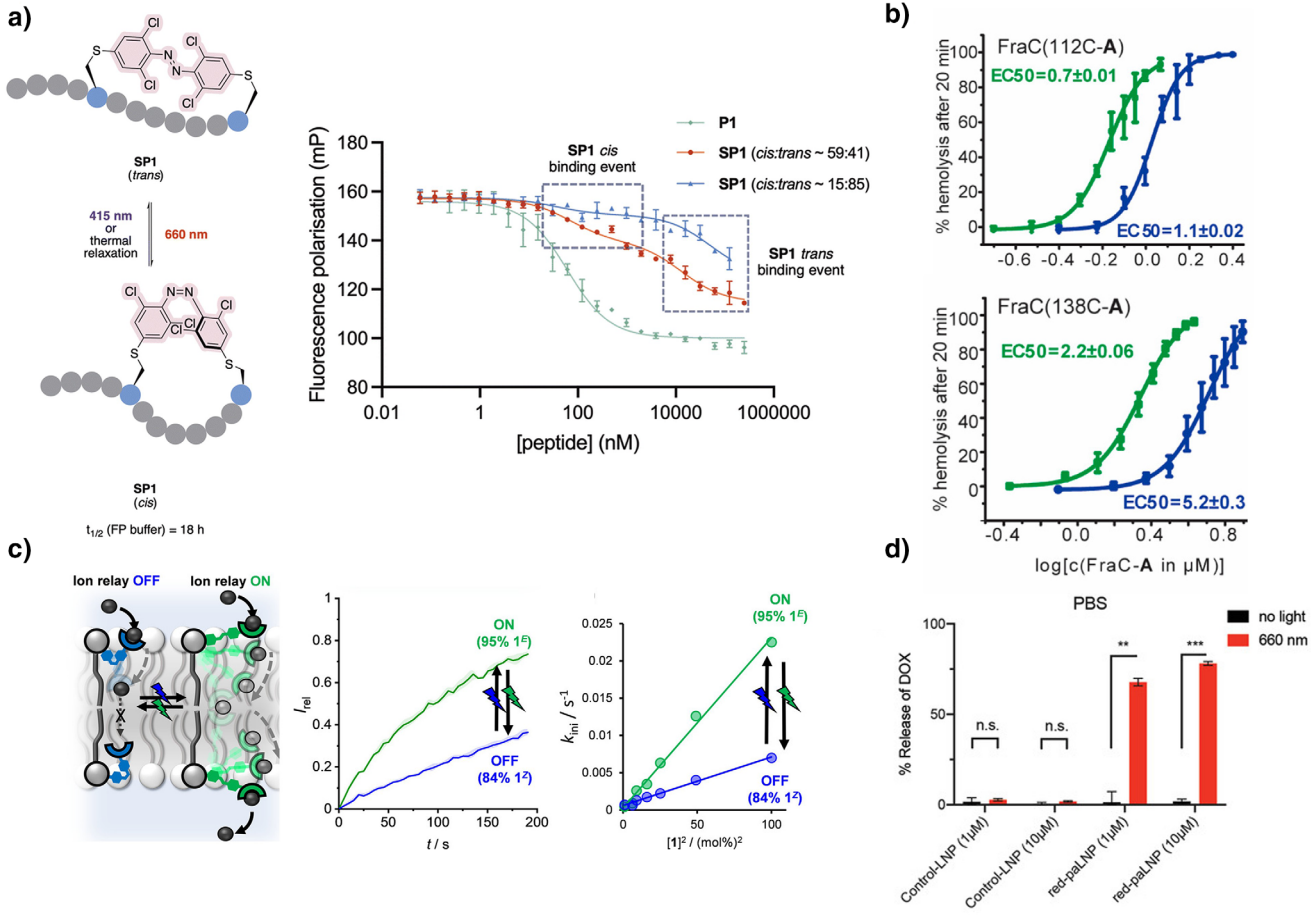
Applications of TOABs in controlling peptides/proteins activities, transport across membranes and drug delivery. a) *E/Z* photoisomerization of the presented stapled peptide (SP1, compound **34** Table 3) and competitive fluorescence polarization assays of *Z* and *E* form of SP1 in respect to a stapled not switchable peptide precursor (P1). Adapted with permission from ref. [98] Copyright © 2024 The Royal Society of Chemistry. b) Hemolytic activity of F_4_TOABs (compound **36**, Table 3) modified FraC under irradiation with 530 (green curves) and 430 nm (blue curves) (EC_50_ values are reported in µM). Adapted with permission from ref. [96] Copyright © 2024 American Chemical Society. c) Representative working mode of the transmembrane transporter and the ion transport evaluation in the two F_4_‐TOAB's isomeric form (compound **37**, Table 3). Adapted with permission from ref. [102] Copyright © 2022 American Chemical Society. d) Evaluation of light‐mediated Doxorubicin release from the liposomal nanocarrier upon pulsed irradiation (660 nm, compound **39** Table 3). Adapted with permission from ref. [105] Copyright © 2021 Wiley‐VCH Verlag GmbH & Co. KGaA.

In this context, the thiol functionality of cysteine (Cys) residues has been frequently used in nucleophilic substitution approaches for linking either F_4_‐TOABs or Cl_4_‐TOABs to peptides or proteins for imparting light control over their activity.^[^ ^96^, ^97^, ^98^ ^]^ The development of photo‐responsive antimicrobial agents has been approached by substituting two amino acids in Tyrocidine A, a cyclic decapeptide with antibacterial activity, with a Cl_4_‐TOAB, inserted by SPPS within the structure to provide a small library of linear and cyclic antimicrobials. In this case, the incorporation of TOABs induced reversible changes in the peptide's β‐hairpin secondary structure upon exposure to visible light at different wavelengths^[^ ^99^ ^]^ (compound **32**, Table 3). Interestingly, **32** showed activity against *A. baumannii* upon exposure to 650 nm light, while becoming inactive upon exposure to sunlight, highlighting the design potentialities and perspectives toward preventing environmental buildup of antibiotic resistance. The photocontrol over cell‐penetrating α‐helical peptide (CPP) has been explored by linking F_4_‐TOAB (compound **33**, Table 3) to Cys residues, inserted in place of lysine ones at positions 7 and 11 in LK_1_ amphiphilic CPP. This design allowed the reversible control over the overall CPP's secondary structure by exposure to 590 or 450 nm light.^[^ ^97^ ^]^ Reversible switching has been successfully achieved and enabled interconverting between a random coil, unorganized structure (*E* isomer) to a more organized amphiphilic helical one (*Z* isomer) without any cytotoxicity evidence, paving the way for further control of drugs or bioactive compounds as interleukins. Peptide stapling and light control in the inhibition of MDM2/p53 protein–protein interactions (PPIs), a key target in cancer therapy, have been studied by linking a Cl_4_‐TOAB (compound **34**, Table 3) to Cys inserted as substituents at positions 5 and 12 of PMI peptide to yield a stapled peptide (SP1).^[^ ^98^ ^]^ The *Z*/*E* switching was reversible and efficient upon irradiation with 660 and 415 nm light, which allowed significantly different binding affinities (Figure 12a) for MDM2: the *Z* isomer showed a 240‐fold stronger affinity in respect to the *E* one upon entire exposure with visible light wavelengths envisioning further precise functional spatiotemporal control and guaranteed stability.

The insertion of F_4_‐TOAB through SPPS into peptide sequences^[^ ^100^ ^]^ (compound **35**, Table 3) was employed to obtain photo‐controlled peptidomimetics capable of inhibiting PPI and ultimately a reversibly photocontrolled inhibition of MLL1, a protein involved in leukemia cell proliferation. Irradiation with 520 nm light enabled the isomerization from *E* to *Z* and 405 nm light resulted in the reverse switching. Glutathione‐Sepharose‐transferase pull‐down experiments revealed how both isomeric forms break the MLL1 complex, with the *E* isomer showing a higher activity. These results are consistent with those previously reported by the same group using a UV‐responsive AB.^[^ ^101^ ^]^ However, in this case, improved performance was achieved by utilizing visible safer light wavelengths with the aim of enabling future in vivo applications.

Finally, Volarić and co‐workers recently linked a water soluble F_4_‐TOAB (compound **36**, Table 3) to a FraC nanopore, a pore‐forming toxin in which two Cys residues were introduced at positions 112 and 138 in the natural sphingomyelin‐binding pocket. These designs aimed at interfering with the nanopore assembly's processes,^[^ ^96^ ^]^ enabling reversible photoswitching between an OFF state (*E* isomer) and an ON one (*Z* isomer) by exposure to 530 nm and 430 nm irradiation, respectively. This resulted in a significantly different cytolytic activity (Figure 12b) in both defibrinated red blood cells (hemolytic activity) and hypopharyngeal squamous carcinoma cells, making it possible to pioneer the control of protein toxicity with visible light.

#### 4.1.3 Light Control of Membrane Transport

The transport across membranes plays a crucial role in controlling biochemical and physiological homeostasis. The possibility of its precise control with visible light provides unique possibilities for engineering the next generation of artificial transport tools of high applicational importance.

A synthetic ion transporter (compound **37**, Table 3) was developed by Johnson and colleagues^[^ ^102^ ^]^ by the incorporation of a F_4_‐TOAB into a phospholipid to develop a visible light photo‐responsive system capable of ion transport through membranes. Specifically, F_4_‐TOAB was functionalized with a terminal aryl‐thiourea, acting as a H‐bond assisted anion binding group. The proper functioning of the artificial transporter was evaluated by analyzing the presence of the anion transporter at both sides of membranes’ leaflet, the transporter length and different membrane composition in large unilamellar vesicles (LUVs). The study of selective transport of different anions was evaluated revealing an ON state with the *E* isomer and an OFF one with the *Z* one (obtained by irradiation with 530 nm light) enabling future application of artificial light‐controlled membrane‐transporters (Figure 12c).

To similarly control anion transport, Cl/F/I/EtS‐TOABs were coupled to mono‐squaramide derivatives to build‐up squaramide‐based membrane anion transporters (compound **38**, Table 3). Those tools could be switched from the OFF state (*E* isomer) to the ON state (*Z* isomer) by irradiation with 625 nm, while the reverted ON‐OFF process was obtained by 455 nm irradiation or exploiting thermal back relaxation. The results obtained provide detailed insights on studying artificial Cl^−^ transport across membranes where the type of *ortho* substituents influence the half‐life of TOABs’ thermal relaxation and consequently the whole transport parameters and the overall application results.^[^ ^103^, ^104^ ^]^

#### 4.1.4 Visible Light‐Controlled Drug Delivery

The photo‐triggered release of drugs offers a unique opportunity for ameliorating drug delivery approaches. The incorporation of TOABs into drug‐carrier systems makes possible the visible light regulated remote control of payloads release.

One attempt toward the light‐triggered drug release focused on the preparation of visible‐light‐responsive liposomal nanocarriers by introducing photoswitchable phospholipids in the liposomal formulation for evaluating the Doxorubicin release.^[^ ^105^ ^]^ The photoswitchable subunits have been prepared by creating esters linkages through reacting COOH functionalities inserted in Cl_4_‐TOABs’ *para* positions and phospholipids’ OH groups (compound **39**, Table 3). The delivery of Doxorubicin was assessed upon the liposome disassembly using pulsed 660 nm LED light during 24 h. This irradiation was responsible for converting the *E* isomer of Cl_4_‐TOAB into the *Z* one that resulted in the overall carrier's disassembly and release of Doxorubicin. Such carrier tools were evaluated both in liver cancer cell lines and zebrafish models showing upon light irradiation excellent Doxorubicin release and comparable activity to the free Doxorubicin, proving successful and complete drug release (Figure 12d). This study highlights the possibility of using TOABs for preparing biocompatible liposomal‐based red‐light photo‐controlled drug delivery tools.

#### 4.1.5 Control of Small Bioactive Compounds

TOABs are prime candidates for incorporation into bioactive molecules, rendering them light responsive along the principles of photopharmacology, which aims to remotely and precisely control the activity of drugs to overcome current limitations of pharmacotherapy, such as poor selectivity, drug resistance and off‐site side effects.^[^ ^3^, ^87^ ^]^ The switching capability of TOABs with visible light wavelengths represent the key step needed for clinical translation in the future.

In their pioneering applications in photopharmacology, both F_4_‐TOAB and Cl_4_‐TOAB were linked to the main scaffold of the antibiotic trimethoprim by nucleophilic substitutions (compound **40**, Table 3)^[^ ^106^ ^]^ to obtain red‐light control over antimicrobial activity. The molecule incorporating F_4_‐TOAB showed significant antibacterial activity in the µM range, with a change in activity upon irradiation with 527 nm light. The *Z* isomer showed a MIC_50_ of 5 µM, as compared to the 10 µM for the *E* isomer in in vitro *Escherichia coli* assays. The compound linked to Cl_4_‐TOAB showed an > 8‐fold enhanced activity (from > 80 µM to 10 µM MIC_50_) upon *E*/*Z* isomerization with 652 nm light, enabling an even more red‐shifted switching for the control of antibacterial activity.^[^ ^106^ ^]^

**Figure 13:**
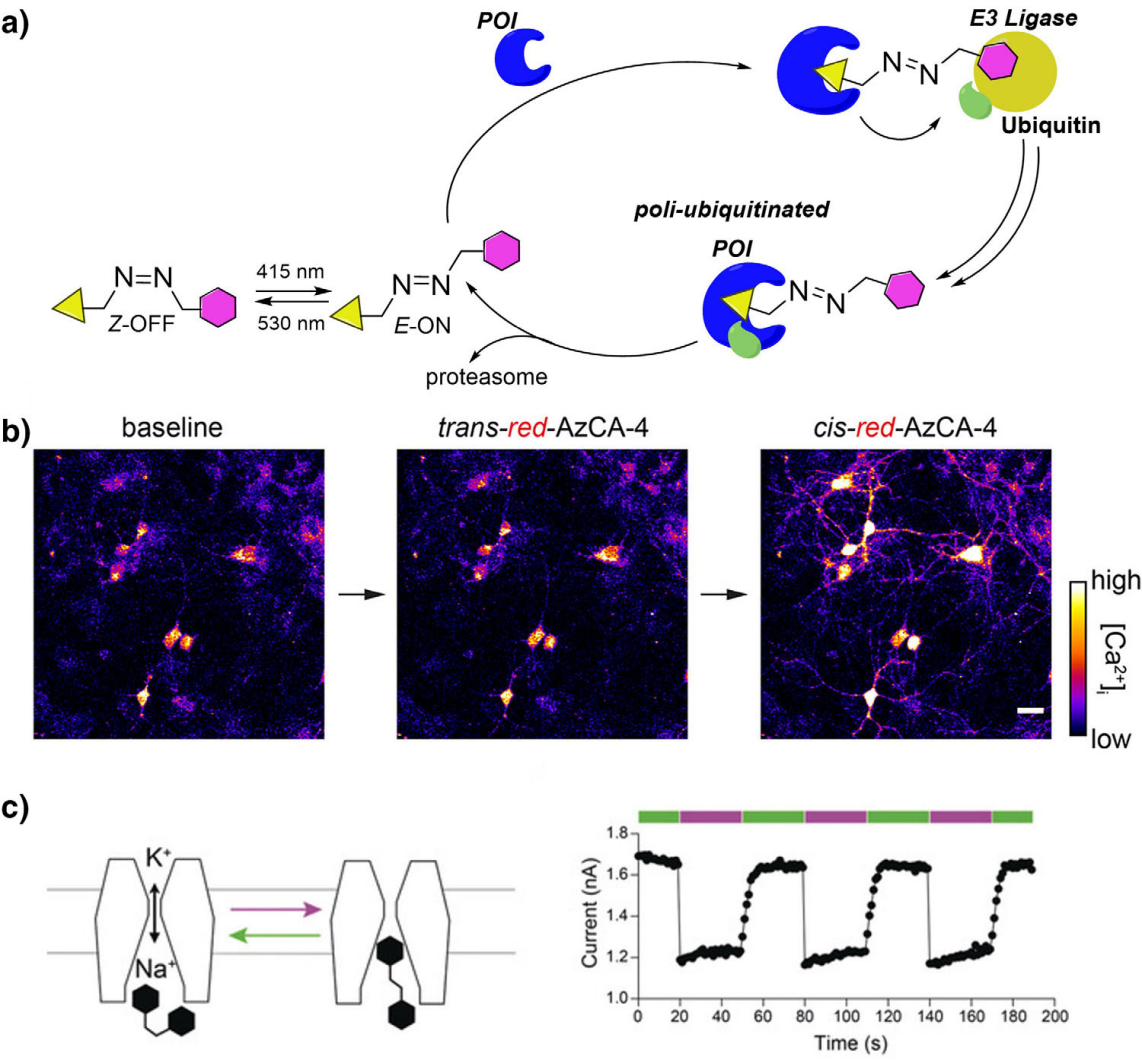
The use of TOABs in photopharmacology. a) The general principle behind photoswitchable PROTACs (compound **41**, Table 3) that use light to modulate interactions between the protein of interest (POI) and E3 Ligase. b) Confocal fluorescence Ca^2+^ imaging in presence of Fluo‐4‐AM dye highlighting the different activity depending on the *E* and *Z* compound **42** (Table 3) isomeric forms (scale bar 25 µm). Adapted with permission from ref. [107] Copyright © 2020 American Chemical Society. c) Representative images of compound **43** (Table 3) *E* isomer being a better channel blocker in respect to the *Z* isomer and the related reversible block of steady state voltage‐gated K^+^ flow upon 533 nm (green bars) and 405 nm (purple bars) light irradiation. Adapted with permission from ref. [108] Copyright © 2018 Wiley‐VCH Verlag GmbH & Co. KGaA.

The introduction of F_4_‐TOAB by amide coupling in Proteolysis Targeting Chimeras (PROTACs), recently led to the next generation of photo‐control in protein degradation tools (compound **41**, Table 3, Figure 13a).^[^ ^79^ ^]^ F_4_‐TOAB worked as a linker allowing to use light to change the length of the compound depending on the AB isomerization state; the longer *E* isomer maintained the pharmacological activity, while in the presence of the *Z* isomer (obtained by irradiation at 530 nm) a shorter structure was obtained and the activity was inhibited. Investigation on Ramos cells have proven the process’ reversibility and the effect of compounds at concentrations in the low nanomolar range, opening new possibilities for visible light photopharmacology in the PROTACs field.

TOABs have also been used in photopharmacological approaches in the field of neuroscience. A modified Cl_4_‐TOAB was used to synthesize a capsaicin analog (compound **42**, Table 3) acting as a visible light‐controlled agonist of TRPV1 receptor to set‐up a multifunctional fiber system usable for in vivo photopharmacology purposes.^[^ ^107^ ^]^ The compound showed higher activity in its *Z* form, which represents the ON state and can be easily obtained by green (500–590 nm) or UV‐A irradiation, while the OFF state, *E* isomer, can be obtained upon exposure to blue light (410–480 nm) (Figure 13b). The experimental setup made of polymeric optical waveguides and two microfluidic channels was used for testing **42** in targeting the ventral tegmental area in mouse brains. This allows the exploration of the mesolimbic pathway in mice, paving the way to the development of new technologies for in vivo photopharmacology and optogenetics studies using light with safe energy.

An ion channel antagonist containing F_4_‐TOAB^[^ ^108^ ^]^ (compound **43**, Table 3) was explored for controlling voltage‐gated ion channels in neurons. The use of the *E* isomer resulted in a better channel blocking as compared to the *Z* one (obtained by 533 nm light irradiation). The exposure to 405 nm light allowed the back switching to the more active *E* form (Figure 13c). Importantly, the compound was also switched in a two‐photon excitation process. Furthermore, a not‐symmetric F_4_‐TOAB (compound **44**, Table 3) was explored as a tool for controlling the activity of acetylcholine dependent receptors channels. The molecule was unsuccessful in a direct channel functionalization for accessing its straightforward light control, but it efficiently worked as a freely diffused probe. This approach enabled green/violet light mediated studies of nicotinic‐acetylcholine receptors in neurons showing an OFF‐state activity in the *E* isomer and an ON‐state in the *Z* one.^[^ ^82^ ^]^

The reported overview on key biological applications highlights the versatility of TOABs and their essential photophysical promise for translating to in vivo applications matching clinical needs. The reported results are in many cases follow‐up studies that improve the applicability of UV‐responsive ABs, as showcased by the works of Albert and colleagues on MLL1 inhibition.^[^ ^100^, ^101^ ^]^ These studies also reveal that it is of key importance to precisely evaluate the ultimate impact of *ortho* substituents on the final biological activity. Those can indeed lead to different biological outcomes in respect to the ones obtained with UV‐responsive ABs. As examples, the results reported by Sansalone^[^ ^82^ ^]^ highlight how the F_4_‐TOAB analog of a ion channel antagonist works only as a free probe, while being much less successful in direct acetylcholine ion channel functionalization as compared to its parent AB analog. In an opposite trend, Agnetta and colleagues show an increased activity for a F_4_‐TOAB modified muscarinic agonist in respect to the AB analog, thanks to a geometry optimization in the target binding due to the presence of F atoms.^[^ ^109^ ^]^

### 4.2 Applications of TOABs in Material Science

The impact of TOABs on material science has recently led to unique opportunities in remote‐control of different constructs for muti‐*stimuli‐*responsive technologies, which are summarized in Table 4 that outlines TOABs scaffolds employed, switching parameters and final targets.

**Table 4:** Overview of TOABs applications in material science.

Entry	Cpd.	Application	Structure	Switching Wavelength^a)^	Ref.
*Polymeric materials*					
*1*	**45**	Molecularly imprinted polymers for Acyclovir release	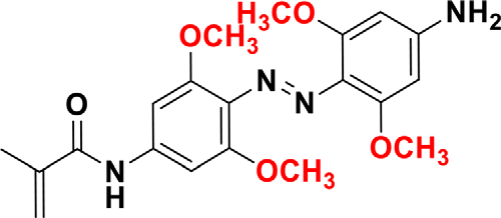	440/630 nm	[110]
*2*	**46**	Amphipilic co‐polymers as carriers	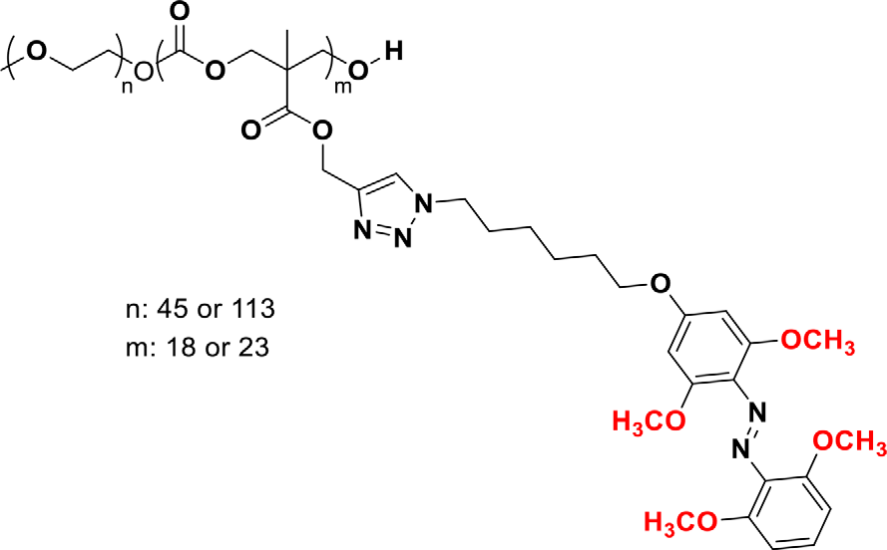	530–625 nm	[111]
*3*	**47**	Polymeric NPs as delivery systems	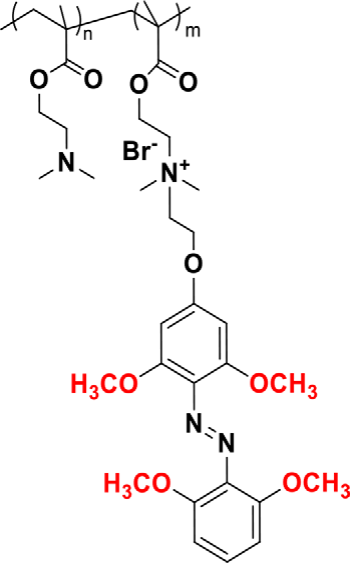	520/450 nm	[78]
*4*	**48**	Terpolymers micelles assembly	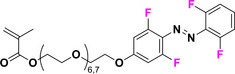	530/415 nm	[83]
*5*	**49**	Triblock‐cyclodextrin polymer‐controlled folding processes	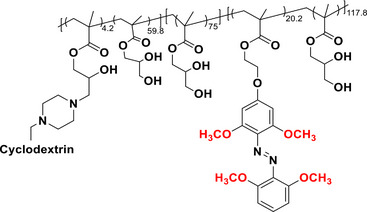	523/470 nm	[112]
*6*	**50**	Liquid crystalline polymeric films/dynamic surfaces	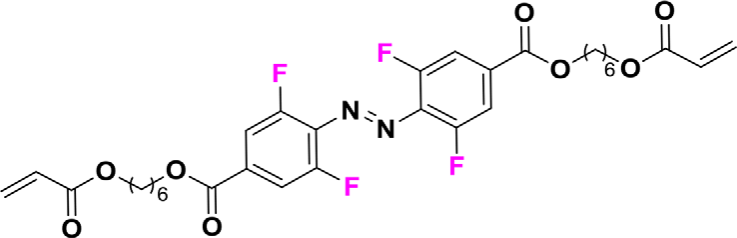	530/405–455 nm	[113, 114]
*Hydrogels and microgels*					
*7*	**51**	Hydrogel for 5(6)‐carboxyfluorescein release	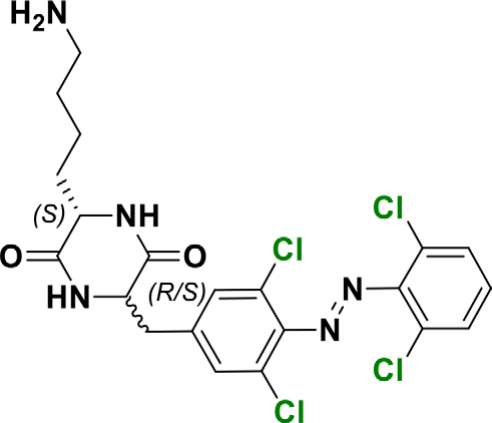	660–523 nm	[115]
*8*	**52**	Host‐guest hydrogel for drug delivery	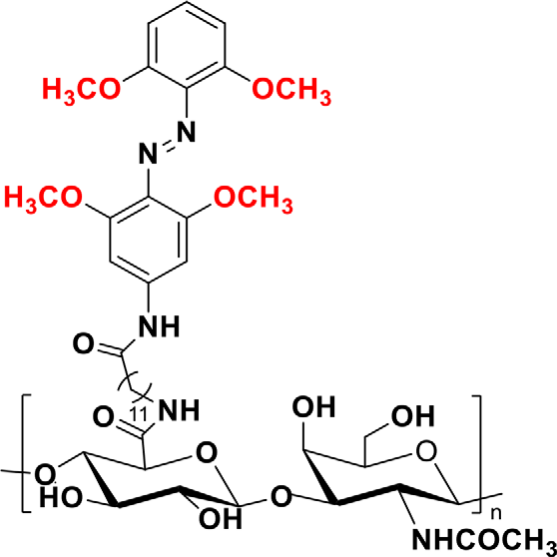	625/470 nm	[116]
*9*	**53**	Hydrogel for payload release	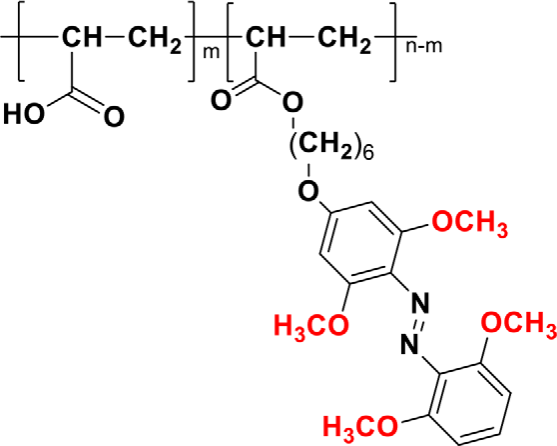	550/450 nm	[117]
*10*	**54**	Microgel for delivery purposes	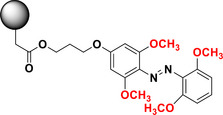	530–625/ 470 nm	[118]
*11*	**55**	Hydrogel with tuneable elasticity	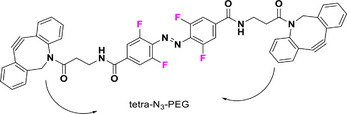	530/405 nm	[119]
*Metal‐organic frameworks*					
*12*	**56**	Core‐Shell MOFs for payload release	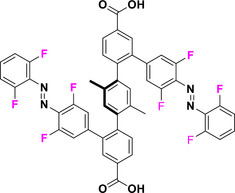	565/420 nm	[120]
*13*	**57**	MOFs for drug release	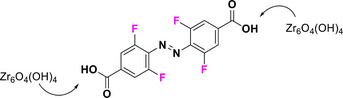	515 nm	[121]
*Supramolecular assemblies/host‐guest systems*					
*14*	**58**	Cucurbitil‐host/guest	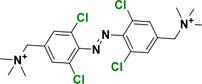	650/410 nm	[122]
*15*	**59**	Cholesteric liquid crystals	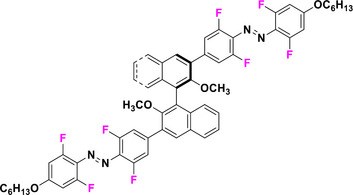	530/450 nm	[77]
*16*	**60**	Dynamic assemblies	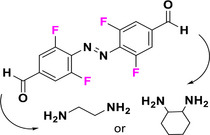	565–660/ 405–470 nm	[123]
*17*	**61**	Halogen bonding receptors	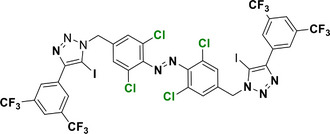	625/455 nm	[124]
*18*	**62**	Co‐crystalline constructs	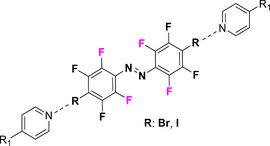	532 nm	[125]

^a)^ Switching wavelengths for *E* →*Z*/*Z* → *E*, respectively. Values given as range highlight two available wavelengths for switching.

#### 4.2.1 Photocontrol of Polymer Structure and Function

The impact of TOABs on different polymeric structures have been widely explored to photocontrol polymer folding properties or the assembly of polymeric NPs and carriers, with a special focus on drug release systems.

**Figure 14:**
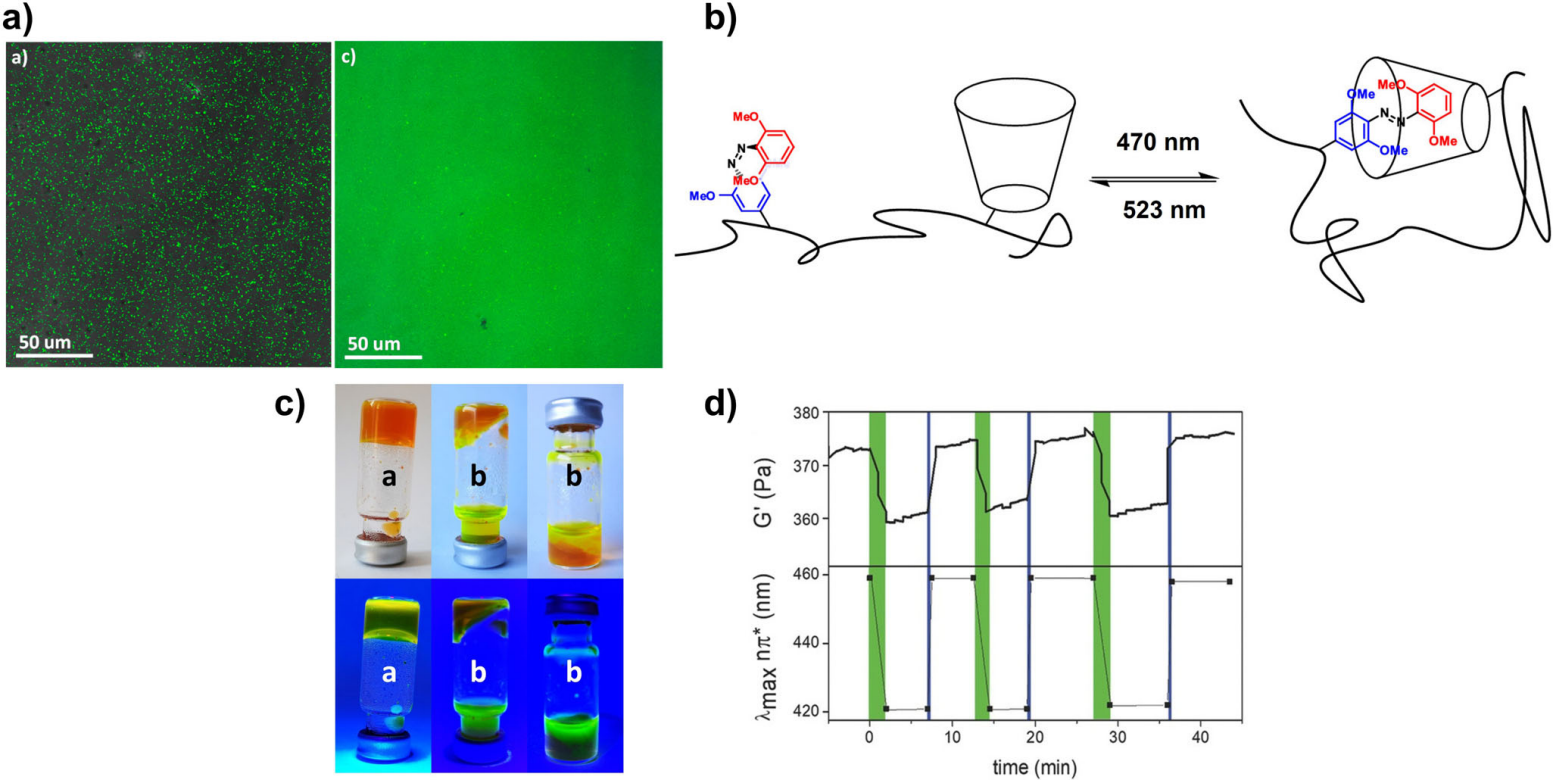
Uses of TOABs in photoswitchable polymers and hydrogels. a) Fluorescence microscopy before (left) and after (right) 530 nm irradiation proving Rhodamine B release upon polymeric particle disassembly upon *E* to *Z* isomerization (**46**, Table 4). Adapted with permission from ref. [111] Copyright © 2022 MDPI. b) Schematic reversible polymeric folding and unfolding phenomena driven by visible light irradiations (**49**, Table 4). Adapted with permission from ref. [112] Copyright © 2021 American Chemical Society. c) a‐Pictures of the hydrogel samples containing carboxyfluorescein at ambient light (top) or under UV irradiation (bottom); b‐the same samples upon 660 nm irradiation, evidencing carboxyfluorescein release from the hydrogel matrix (**51**, Table 4). Adapted with permission from ref. [115] Copyright © 2023 Wiley‐VCH Verlag GmbH & Co. KGaA. d) Simultaneous variation of storage modulus (G’) and photoisomerization upon 530 nm irradiation obtained by incorporating compound **55** into hydrogel matrixes. Adapted with permission from ref. [119] Copyright © 2018 Wiley‐VCH Verlag GmbH & Co. KGaA.

The light‐dependent release of antiviral agents (Acyclovir) has been studied by taking advantage of the unique switching properties of (MeO)_4_‐TOAB for preparing visible light responsive surface molecularly imprinted polymer (VSMIP) for coating SiO_2_ NPs.^[^ ^110^ ^]^ The photo‐responsive unit (compound **45**, Table 4) switches from the *E* to the *Z* isomer with irradiation at 440 nm causing the release of Acyclovir, while the irradiation with 630 nm (*Z* to *E* isomerization) allowed the uptake within the VSMIP of the previously released Acyclovir. The ex vivo experiments were also successful with chicken skin tissues, proving deep tissue penetration capability and application of TOAB‐based visible light mediated drug‐release systems. Other carrier systems have been studied in light‐controlled assembly as vesicles or micelles in water by combining amphiphilic linear di‐block copolymers made of poly(ethyleneglycol) portions and (MeO)_4_‐TOAB inserted using click chemistry (compound **46**, Table 4).^[^ ^111^ ^]^ Irradiation with 625 nm or 530 nm light enabled successful TOAB's *E* to *Z* isomerization, impacting on the overall particles properties. Exposure to 530 nm wavelength resulted in vesicle disassembly and model payloads (Rhodamine B and Nile Red) release, opening possibility to designing novel visible light responsive nano‐carriers (Figure 14a).

Other versatile multi‐responsive polymeric NPs capable of micellar assembly in water have been prepared by using (MeO)_4_‐TOAB combined with poly(dimethylaminoethyl methacrylate) (compound **47**, Table 4).^[^ ^78^ ^]^ The NPs were tested for responsivity to light (520 nm), pH and ß‐cyclodextrin (ß‐CD) in the release of a model payload, Nile Red. The switching from *E* to *Z* isomer (under 520 nm light) showed limited release of the payload, while acidic pH or ß‐CD was responsible for payload release by swelling or dissociation effects, respectively, providing significant insight for further development of biotechnological light responsive platforms. Novel light‐responsive micellar constructs have been evaluated by synthesizing water soluble polymer made of three different monomer subunits (terpolymer) containing a F_4_‐TOAB (compound **48**, Table 4). This polymer has been evaluated for its self‐assembly behavior in water, additionally exploring the terminal maleimide functionality for further bioconjugation or in hydrogel formation, which however resulted in an inefficient translation of the light responsivity occurring at the molecular level to the materials.^[^ ^83^ ^]^

The visible light control toward high molecular weight polymer folding has been studied by the preparation of a multi‐stimuli responsive folding triblock polymer containing ethylene glycol monomers, (MeO)_4_‐TOAB units (compound **49**, Table 4) and ß‐CD moieties to evaluate light control on the polymeric folding processes between the two latter components (Figure 14b).^[^ ^112^ ^]^ The presence of (MeO)_4_‐TOAB enabled the light control: the *E* to *Z* isomerization occurring with green light (523 nm) was responsible for unfolding phenomena, while exposure to blue light (470 nm) revert back the (MeO)_4_‐TOAB to the *E* form resulting in successful folding in a reversible manner. Further evaluation focused on the self‐inclusion property of the polymer linked (MeO)_4_‐TOAB and ß‐CD for its use as a visible light controlled functional platform.

The key properties of F_4_‐TOAB (compound **50**, Table 4) were instrumental in the design of new liquid crystalline‐polymeric films^[^ ^113^ ^]^ and materials acting as visible responsive dynamic surface topographies.^[^ ^114^ ^]^ These constructs behave as soft actuators capable of structural self‐oscillation when exposed to sunlight. To evaluate the functioning at specific wavelengths, the prepared material was exposed to LED light at 530 nm and 405 nm to monitor in detail the F_4_‐TOAB *E* to *Z* and *Z* to *E* isomerization and the related polymeric structural variations. A flat shape is observed in the presence of *Z* F_4_‐TOAB obtained upon green light exposure, while a bent polymeric structure relates to the *E* F_4_‐TOAB isomeric form re‐obtained upon blue light irradiation.^[^ ^113^ ^]^ These artificial constructs behave indeed like natural materials that use sunlight as a continuous energy source. To confirm their potential, further investigations using 455 nm as an alternative blue light wavelength (much more suitable for applications as compared to the 405 nm one) were conducted to evaluate the responsive topographies as surface for cells growth.^[^ ^114^ ^]^ In the assay, the NIH/3T3 fibroblasts maintained their living status while they changed their shapes depending on the light irradiation wavelengths, proving the biocompatibility and applicability of the F_4_‐TOAB‐based smart surfaces.

#### 4.2.2 Visible Light Control of Hydrogels and Microgels

The studies of the next generation of materials for (bio)technological purposes has recently focused on combining the intrinsic chemical‐physical properties of hydrogels with TOABs’ visible light switching capabilities to investigate new materials capable of tuning intrinsic gel properties, allowing controlled release and various hosts‐guests interactions.

The combination of Cl_4_‐TOAB with cyclic dipeptides as gelator building blocks was studied to develop hydrogel systems capable of efficient red‐light mediated release of drugs and biopolymers under physiological conditions (compound **51**, Table 4)^[^ ^115^ ^]^ The exposure to 660 nm light switches TOAB **51** from its *E* to *Z* isomer, resulting in significant hydrogel shrinking. Heating was required for obtaining the initial hydrogel texture, because the exposure to 455 nm light, although capable of restoring the *E* isomer, was not efficient for obtaining the initial texture. The hydrogel was tested in the successful red‐light‐only‐controlled cargo release of carboxyfluorescein with a negligible cytotoxicity, paving the way of further biomedical uses of such hydrogel/syringe‐like set‐ups (Figure 14c).

Smart materials for multiple purposes, including self‐healing, reversible adhesion and protein release in a drug delivery approach, have been created by engineering supramolecular semi‐switchable hydrogel systems. This goal was achieved by incorporating (MeO)_4_‐TOAB and ß‐CD as host/guest units in a hyaluronic acid‐based supramolecular structure (compound **52**, Table 4).^[^ ^116^ ^]^ Upon exposure to 625 nm light (resulting in *E* to *Z* isomerization), host/guest interactions between (MeO)_4_‐TOAB and ß‐CD disappeared and could be restored upon 470 nm light irradiation. Moreover, the system showed semi‐convertible sol‐gel transition upon 625 nm light irradiation, showcasing light‐modulation of microscopic dynamics and simultaneous macroscopic stability broadening the applications in material bioengineering thanks to their proven cytocompatibility and manufacturability. Similarly, a multiple stimuli‐responsive hydrogel has been created by combining (MeO)_4_‐TOAB and ß‐CD subunits (compound **53**, Table 4) to evaluate its use as a visible light‐sensitive drug delivery platform.^[^ ^117^ ^]^ In this case, besides temperature and pH responsivity, the presence of TOAB and its irradiation impacts the host/guest interactions between TOABs and ß‐CD. When the hydrogel is irradiated with 550 nm light (*E* to *Z* isomerization), its mechanical strength decreases, leading to the sol form due to gel phase decomposition. In contrast, upon irradiation with 450 nm light, the TOAB's *E* form was restored, regenerating a gel structure. This reversible phenomenon was successfully employed for the Rhodamine B payload release enforcing the potentiality of these hydrogel design for biomedical materials.

Poly(*N*‐vinylcaprolactam‐co‐acrylic acid) microgels containing (MeO)_4_‐TOAB have been used to create a pH, temperature and light‐responsive microgel with possible applications such as drug delivery or light sensor tools. (compound **54**, Table 4).^[^ ^118^ ^]^ The microgel NPs’ size and hydrodynamic radius measured at pH 8 were significantly changed upon green or red‐light irradiation, responsible of *Z* isomer formation. Under these conditions, swelling effects were observed for the *Z* isomer (green/red‐light). Conversely, shrinking effects occurred for the *E* isomer when irradiated with blue light, due to significant polarity changes.

The development of visible light controlled supramolecular machines has been approached by an experimental and computational investigation aimed at understanding the photo‐modulation of the overall hydrogel's elasticity related to the TOAB switching process.^[^ ^119^ ^]^ To this end, F_4_‐TOABs have been incorporated, using click chemistry, into a PEG‐based hydrogel (compound **55**, Table 4). *E* to *Z* isomerization occurred under 530 nm light irradiation, while the reverse process could be achieved with 405 nm light. The shear elastic modulus and the related storage modulus (G’) decreased upon the *E* to *Z* isomerization, while the reverse *Z* to *E* isomerization resulted in restoration of the initial elasticity, in a reversible manner and with good fatigue resistance (Figure 14d). The study showed how the overall variation in elasticity of these hydrogels’ type seems dependent on the extra cross‐linker role played by the stable *E* isomer that is disrupted upon *E* to *Z* isomerization imparting unique elasticity traits and further applicational possibilities of those smart materials.

#### 4.2.3 Control of Metal Organic Frameworks (MOFs)

**Figure 15:**
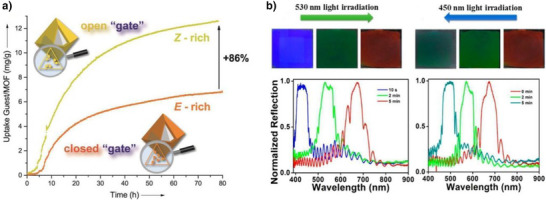
Photoresponsive MOFs and cholesteric liquid crystals containing TOABs. a) Uptake studies for 1‐pyrenecarboxylic acid which proves the (compound **56** Table 4) *E*‐isomer‐rich MOF (close‐gate/lower guest uptake) in respect to the *Z‐*isomer‐rich one (open‐gate/higher guest uptake). Adapted with permission from ref. [120] Copyright © 2019 Wiley‐VCH Verlag GmbH & Co. KGaA. b) Images of the cholesteric liquid crystals and reflection spectra in planar cells upon 530 nm (left) or 450 nm (right) irradiation, highlighting color tuning upon irradiation and compound **59** (Table 4) isomerization. Adapted with permission from ref. [77] Copyright © 2019 American Chemical Society.

TOABs have been recently proposed to confer visible light responsivity upon MOFs, allowing their use as constructs for green light driven multiple cargo release. Hecht and co‐workers used a cross coupling reaction to assemble a two‐component core‐shell zirconia‐MOFs characterized by an internal cavity suitable for guest storage, and a photoswitchable external shell, where F_4_‐TOAB act as a visible‐light‐controlled gate for uptake and release of guest molecules (compound **56**, Table 4).^[^ ^120^ ^]^ Several studies were conducted with different guests (1‐pyrenecarboxylic acid, methylene blue, and 8‐hydroxypyrene‐1,3,6‐trisulfonic acid trisodium salt) showing how the presence of F_4_‐TOAB enables the formation of a reversible kinetic gate that selectively controls the uptake. The uptake was slower for the *E*‐rich MOF (close‐gate) in comparison to the *Z‐*rich one (open‐gate), which is obtained upon exposure to 565 nm light (Figure 15a). Similarly, the guest release has been achieved by introducing F_4_‐TOABs into zirconia‐MOFs backbones (compound **57**, Table 4).^[^ ^121^ ^]^ The release of 5‐fluorouracil (5‐FU) was studied on an amino‐PEG modified MOFs; the PEG modification was necessary to assess proper stability in aqueous environment. The 5‐FU release was studied by irradiating the sample with 515 nm light to obtain the *Z* isomer responsible of a significant cargo release and coherent cytotoxicity toward colorectal cancer cell lines, as compared to the non‐irradiated form corresponding to the TOAB's *E* isomer. Moreover, the green light exposure resulted in a spontaneous photo‐exfoliation of the whole MOF structure, paving the way to applications for significant control of cargo release accompanied by a rapid degradation of the delivery subunit.

#### 4.2.4 Visible Light Control of Supramolecular Assembly/Host‐guest Systems

Visible light control over different types of host‐guest systems offers unique possibilities for precise, on‐demand control of specific interactions as well as for tuning host‐guest relations. Host‐guest systems release have been realized by combining Cl_4_‐TOABs‐guests (compound **58**, Table 4) and cucurbit[8]uril‐hosts to develop biocompatible photoresponsive supramolecular systems for the light‐controlled release of co‐cargo moieties.^[^ ^122^ ^]^ Irradiation with red light (615 nm) resulted in *E* to *Z* isomerization of Cl_4_‐TOABs, allowing the concomitant release of both water‐soluble and water‐insoluble pharmaceutically active compounds, such as capecitabine or tamoxifen, providing insights for further co‐guests light‐controlled release of drugs.

Chiral, visible light responsive constructs containing F_4_‐TOABs (compound **59**, Table 4) have been studied for preparing cholesteric liquid crystal systems. The TOAB's *E* to *Z* isomerization was achieved under 530 nm light irradiation, while the reverse process occurred under 450 nm light.^[^ ^77^ ^]^ The light responsiveness could be translated into a remarkable change in helical twisting power, which allowed significant circularly polarized reflection colors at different wavelengths (Figure 15b).

Furthermore, imine macrocycles have been developed by incorporating F_4_‐TOABs and diamine moieties for providing access to dissipative/metastable dynamic covalent systems regulated by visible light (compound **60**, Table 4).^[^ ^123^ ^]^ In the TOAB/ethylenediamine combinations, only the *Z* isomer of the F_4_‐TOAB, obtained by irradiation with 660 nm light, gave access to polymeric macrocycles, while the *E* isomer was responsible for formation of insoluble polymers. Moreover, other macrocyclic assemblies, where a cyclic amine (1,2‐cyclohexanediamine) was employed, underwent light‐dependent contraction and extension processes, opening possibilities for studying out of equilibrium materials.

Finally, anions receptors, in which halogen bonding can be controlled by light, have been prepared by introducing Cl_4_‐TOABs into the molecular receptor scaffold (compound **61**, Table 4).^[^ ^124^ ^]^ The affinity toward chloride anion was precisely controlled by light: the *Z* isomer (obtained upon irradiation at 625 nm) showed up to > 50 greater affinity as compared to the *E* isomer (obtained upon re‐irradiation at 455 nm or by heating). Such tools enabled successful light control over the catalysts in Murayama‐aldol and Friedel‐Crafts alkylation reactions, exploring TOABs’ possibilities for light control of catalytic processes or molecular sensing.

Finally, halogen‐bond mediated co‐crystallization of F_4_‐TOAB, featuring Br or I as substituents in *para* positions (compound **62**, Table 4), has been explored for preparing co‐crystalline constructs. These assemblies show different geometries depending on the *Z* or *E* TOABs isomeric forms interacting with the pyridine counterparts to yield the final co‐crystal.^[^ ^125^ ^]^ They were studied by X‐ray crystallography, showing different, visible‐light‐dependent photo‐mechanical motions, useful toward the preparation of new materials with intrinsic photomechanical behaviors.

**Figure 16:**
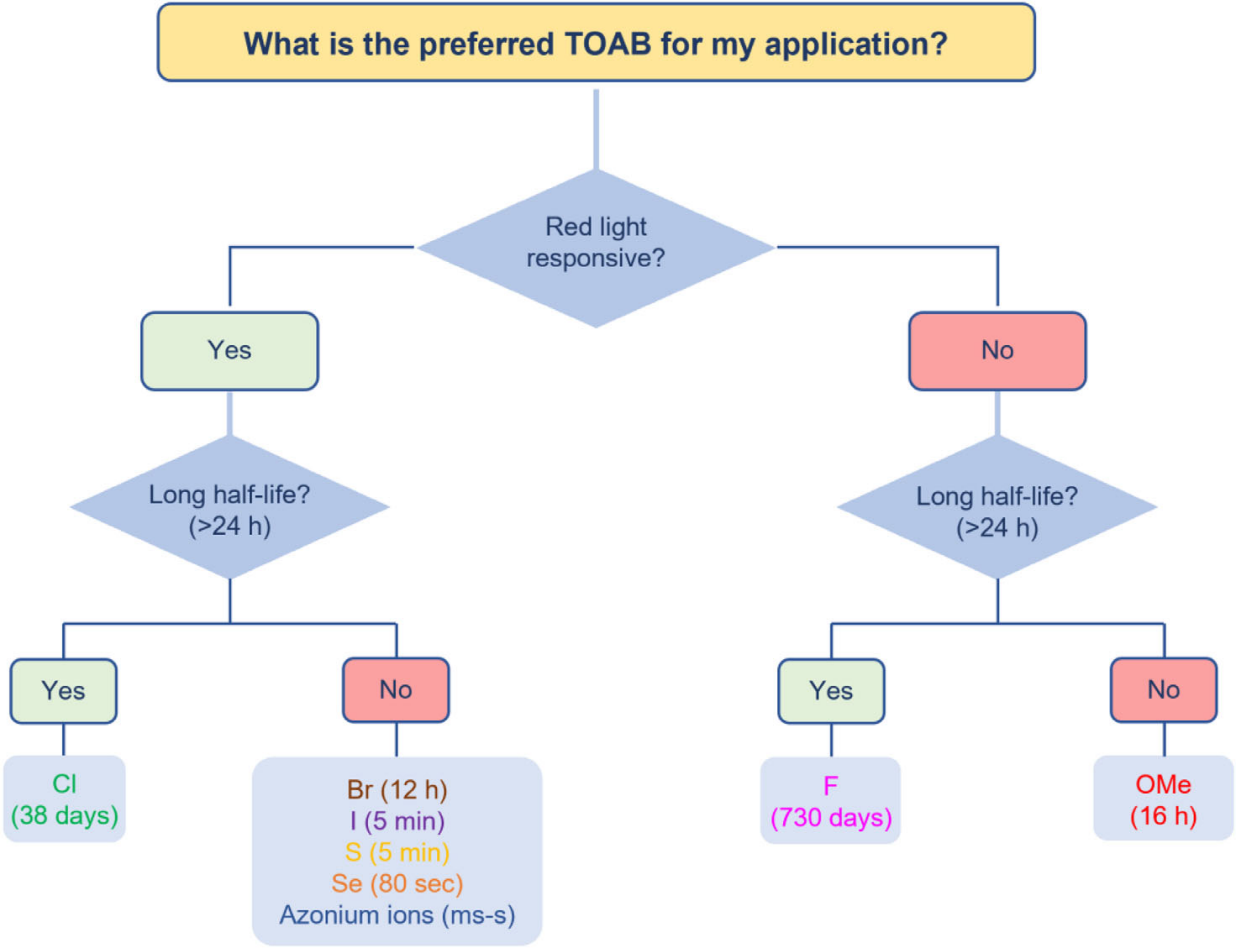
Flow chart guiding the selection of the most suitable TOAB for biological and materials applications based on its photochemical properties and visible‐light responsiveness.

The versatility of TOABs has been showcased in many applications within the last decade. This overview highlights how F_4_‐TOABs, Cl_4_‐TOABs and (MeO)_4_‐TOABs are currently the most frequently used TOABs. However, selecting the most suitable TOAB for a specific application can be challenging. To facilitate this process, Figure 16 presents a flow chart designed to guide the reader in making an informed choice based on the photochemical properties of the TOAB derivatives.

## 5 Summary and Outlook

TOABs constitute a class of visible‐light‐responsive photoswitches that show unique optical properties while maintaining a compact molecular structure. Additionally, they offer a high degree of versatility in terms of substituents and functionalization patterns, enabling a plethora of applications in several research areas.

Overall, TOABs enable a broader range of applications compared to other visible/NIR light responsive photoswitches. Although recent studies have demonstrated promising results for the alternative azoheteroarenes in certain applications, TOABs remain a more reliable choice due to their extensively characterized and well‐established properties within the scientific community. Moreover, TOABs present more tunable photochemical properties, providing enhanced versatility and control in a wider range of applications compared to azoheteroarenes.^[^ ^19^ ^]^ Furthermore, TOABs provide substantially broader functionalization possibilities that are synthetically accessible, unlike diazocines, where the synthesis of non‐symmetric derivatives is considerably more complex.^[^ ^19^ ^]^ When compared to Azo‐BF₂ switches, TOABs exhibit greater stability against hydrolytic processes, since Azo‐BF₂ photochroms have the tendency to revert to hydrazones in aqueous systems.^[^ ^20^ ^]^ In contrast to donor‐acceptor Stenhouse adducts (DASAs), the small and compact structure of TOABs facilitates their application in chemical biology and photopharmacology, whereas the intrinsic molecular structure of DASAs and their incompatibility with aqueous solutions renders their integration significantly more challenging.^[^ ^24^ ^]^

At the same time, TOABs are still facing several drawbacks, including rather poor water solubility and limited synthetic accessibility and often suboptimal photochemical performance. Many of the explored synthetic routes require long reaction times or involve hazardous reagents and harsh conditions. Moreover, the handling and purification of these compounds can be demanding, posing additional difficulties. Thus, it is urgent to develop new sustainable and accessible synthetic methods that yield high‐purity products, facilitating their use in both research and scaled‐up industrial applications

Furthermore, TOABs show some photochemical limitations, especially lack of response to near‐infrared (NIR) light, which restricts their utility in applications requiring deep tissue penetration. Additionally, strategies used to bathochromically shift their absorption spectra often result in shortening of the *Z* isomer half‐life, compromising the overall photophysical properties. Although azonium ions feature higher absorptivity in the red‐light spectral region, they exhibit poor dynamic ranges in photochemical responses, restricting their versatility in applications needing substantial variations in response to light stimulus. Moreover, the inherent stability issues faced by azonium ions further complicate their use in practical settings.

To address these limitations, the scientific community should prioritize the development of innovative synthetic strategies that enhance the yield, scalability and environmental accessibility of TOABs while also improving the dynamic range of azonium ions. Additionally, efforts should be directed toward optimizing the photochemical properties of both classes of compounds to maximize their efficiency and functionality without compromising stability. Of note, various strategies have been developed to enhance the water solubility of azobenzenes^[^ ^17^ ^]^ for biological applications, which, while not being specific to TOABs, can also be applied to them. By collaboratively advancing research in these areas, the full potential of TOABs and azonium ions can be unlocked, expanding their applicability across an even broader range of fields and ultimately leading to novel solutions and applications that harness their unique characteristics.

## Conflict of Interests

The authors declare no conflict of interest.

## Data Availability

Data sharing is not applicable to this article, as no new data were created or analyzed in this study.
